# SV2B Promotes the Progression of TFE3‐Rearranged Renal Cell Carcinoma by Interacting with HERC2 to Impede the Degradation of NF‐κB Subunits

**DOI:** 10.1002/advs.202509443

**Published:** 2025-11-06

**Authors:** Huayi Feng, Zhuang Xiong, Shaoqing Yu, Junxiao Liu, Shouqing Cao, Kan Liu, Xing Huang, Lequan Wen, Baojun Wang, Qingbo Huang, Tiejun Pan, Xu Zhang, Xin Ma, Xiubin Li

**Affiliations:** ^1^ Department of Urology The Third Medical Center Chinese PLA General Hospital Beijing 100039 China; ^2^ Department of Urology General Hospital of Central Theatre Command Wuhan 430070 China; ^3^ Department of Urology The First Affiliated Hospital of Zhengzhou University Zhengzhou 450000 China

**Keywords:** HERC2, NF‐κB, padsevonil, SV2B, TFE3‐rearranged renal cell carcinoma, Withaferin A

## Abstract

TFE3‐rearranged renal cell carcinoma (TFE3‐RCC) represents an aggressive subtype of renal cancer characterized by a poor prognosis, yet lacking clearly defined diagnostic and therapeutic targets. Here, the study demonstrates that synaptic vesicle protein 2B (SV2B) is a potential diagnostic marker and therapeutic target for TFE3‐RCC. *SV2B*, identified as a TFE3 target gene, is significantly upregulated in TFE3‐RCC and displays high diagnostic accuracy for this subtype. Functionally, SV2B enhances the proliferation, migration, and invasion of TFE3‐RCC cells. Mechanistically, SV2B competes with RELA/NFKB1 for binding to the E3 ligase HERC2, preventing their degradation and activating the NF‐κB pathway. The drug padsevonil, targeting SV2B, selectively inhibits the growth of TFE3‐RCC cells and organoids, similar to the NF‐κB inhibitor Withaferin A. Clinically, RELA/NFKB1 expression positively correlates with SV2B levels but negatively with HERC2 expression, confirming the activation of TFE3‐SV2B‐NF‐κB axis in TFE3‐RCC. These findings establish SV2B as a novel diagnostic biomarker for TFE3‐RCC and validate SV2B‐NF‐κB signaling as a therapeutic target, providing potential strategies for managing TFE3‐RCC.

## Graphical abstract

TFE3‐rearranged renal cell carcinoma (TFE3‐RCC) lacks clearly defined diagnostic and therapeutic targets. SV2B, a TFE3 target gene, promotes TFE3‐RCC progression by activating the NF‐κB pathway. Targeted inhibition with padsevonil or Withaferin A effectively suppresses tumor progression. This study establishes SV2B as a novel diagnostic biomarker and validates SV2B‐NF‐κB signaling as a therapeutic target for TFE3‐RCC.

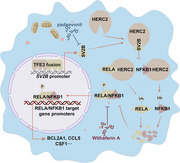

## 1 Introduction

Renal cancer is a prevalent urologic malignancy, with an annual incidence of 0.43 million and an annual mortality of 0.15 million.^[^ ^1^ ^]^ *TFE3*‐rearranged renal cell carcinoma (TFE3‐RCC) is a highly aggressive subtype with limited treatment options in advanced stages, resulting in poor prognosis.^[^ ^2^, ^3^, ^4^, ^5^ ^]^ Moreover, TFE3‐RCC is often misdiagnosed as kidney renal clear cell carcinoma (KIRC) or kidney renal papillary cell carcinoma (KIRP).^[^ ^6^, ^7^ ^]^ because of a lack of reliable diagnostic markers.^[^ ^8^, ^9^ ^]^ Therefore, accurate diagnostic methods and therapeutic targets for TFE3‐RCC are urgently needed.

TFE3‐RCC is generally diagnosed based on immunohistochemical (IHC) analysis of markers such as TFE3, MLANA, HMB45, and CTSK.^[^ ^7^, ^10^, ^11^ ^]^ However, these markers do not always reliably distinguish TFE3‐RCC from KIRC and KIRP.^[^ ^8^, ^9^ ^]^ GPNMB and TRIM63 have been recently identified as potential biomarkers for TFE3‐RCC, although some KIRC and KIRP cases also stain positive for these markers.^[^ ^12^, ^13^ ^]^ We previously identified nicotinamide riboside kinase 2 (NMRK2) as a potential indicator of TFE3‐RCC;^[^ ^14^ ^]^ however, further validation in clinical trials is needed. Hence, new, efficient biomarkers for TFE3‐RCC are urgently required.

Because of the limited treatment options available for advanced TFE3‐RCC, standard therapies developed for KIRC have been used, but with varying outcomes.^[^ ^2^, ^15^, ^16^, ^17^, ^18^ ^]^ Alternative drugs, such as cabozantinib (a MET inhibitor),^[^ ^19^, ^20^ ^]^ may be effective for some but not all TFE3‐RCC patients, as MET expression levels vary. TFE3 functions as a master transcription factor that regulates cellular growth and survival and orchestrates diverse biological processes essential for maintaining tissue homeostasis. By coordinating lysosomal biogenesis, autophagy modulation, and metabolic adaptation, it safeguards physiological integrity across organs.^[^ ^21^ ^]^ Suppressing tumor progression through inhibition of TFE3 irreversibly compromises survival‐essential pathways in healthy tissues, eliciting substantial treatment‐related toxicity. This broad‐spectrum functional impairment profoundly constrains the clinical translational potential of TFE3‐directed therapies. Therefore, targeting genes downstream of TFE3 may be a safer approach for advanced TFE3‐RCC. We previously identified NMRK2 as a direct target of TFE3 fusion proteins; however, effective drugs targeting NMRK2 are lacking.^[^ ^14^, ^22^ ^]^ Further exploration of other TFE3 target genes involved in TFE3‐RCC progression is necessary.

SV2B, a synaptic vesicle protein 2 (SV2) family member, acts as a redundant Ca^2+^ regulator in neurotransmitter release.^[^ ^23^, ^24^ ^]^ Lack of SV2B protein can disrupt synaptic circuits and lead to epilepsy.^[^ ^23^, ^24^ ^]^ Elevated SV2B levels have been associated with poor prognosis in glioblastoma and uterine corpus endometrial cancer.^[^ ^25^, ^26^ ^]^ However, its potential as a diagnostic marker for TFE3‐RCC and biological function in tumors, particularly in TFE3‐RCC, remains to be elucidated.

In the present study, we identified *SV2B* as a direct target gene of TFE3 that is specifically upregulated in TFE3‐RCC. Diagnostic methods based on SV2B expression levels were established for identifying TFE3‐RCC. Functionally, SV2B was found to promote the proliferation, migration, and invasion of TFE3‐RCC cells. Mechanistically, it competes with RELA/NFKB1 for binding to the HECT domain of HERC2, inhibiting RELA/NFKB1 degradation via the ubiquitin‐proteasome pathway, leading to NF‐κB signaling pathway activation. Padsevonil and Withaferin A were found to selectively inhibit the progression of TFE3‐RCC cells and organoids. RELA/NFKB1 expression was positively correlated with SV2B expression, and negatively with HERC2 expression in TFE3‐RCC patients. TFE3‐SV2B‐NF‐κB axis activation was confirmed in TFE3‐RCC. This study identified SV2B as an efficient biomarker for TFE3‐RCC and highlighted the role of the TFE3‐SV2B‐NF‐κB axis in TFE3‐RCC progression, providing new clinical diagnostic and treatment strategies for the disease.

## 2 Results

### 2.1 SV2B is an Effective Distinctive Diagnostic Marker for TFE3‐RCC

**Figure 1:**
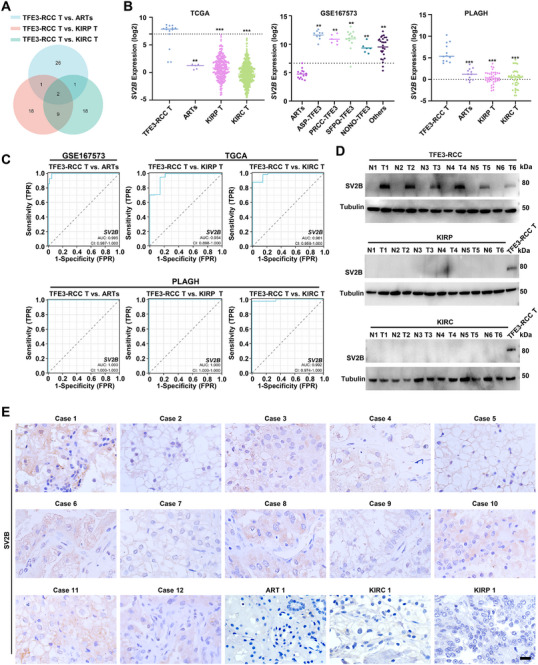
SV2B is an effective diagnostic marker for distinguishing TFE3‐RCC. A) The top 30 differentially expressed genes among the groups were shown in the Venn diagram. *SV2B* and *NMRK2* were the two most prominently expressed genes in TFE3‐RCC tissues. B) Compared to TFE3‐RCC tissues, *SV2B* expression in ARTs, KIRP, and KIRC tissues were significantly lower using the TCGA database and our cohort. Compared to ARTs, *SV2B* expression in TFE3‐RCC with ASP‐TFE3, PRCC‐TFE3, SFPQ‐TFE3, NONO‐TFE3, and other fusion genes were significantly higher using the GEO database. C) Sensitivity and specificity of *SV2B* for distinguishing TFE3‐RCC from ARTs, KIRP, and KIRC tissues using public databases and our cohort, respectively. D,E) SV2B protein expression was explored in TFE3‐RCC, ARTs, KIRP, and KIRC tissues in our cohort. These experiments were replicated three times. Data are presented as the mean ± SD. Scale bars =20 µm; **p < 0.01; ***p < 0.001.

Differential gene expression analysis of TFE3‐RCC tissues and adjacent renal tissues (ARTs), as well as KIRC and KIRP tissues, revealed that *SV2B* and *NMRK2* were the most highly expressed genes in TFE3‐RCC (**Figure** **1**A; Tables S1–S3, Supporting Information). We previously identified NMRK2 as an efficient diagnostic indicator of TFE3‐RCC;^[^ ^14^ ^]^ therefore, in the present study, we focused on SV2B.

SV2B was evaluated as a potential diagnostic marker for TFE3‐RCC by examining its expression levels in TFE3‐RCC, KIRC, and KIRP. Elevated expression of SV2B in TFE3‐RCC tissues was confirmed in public databases and our cohort (Figure 1B; Table S4, Supporting Information). *SV2B* expression levels were higher specifically in TFE3‐RCC tissues than those of *TFE3*, *MLANA*, *HMB45*, and *CTSK* (Figure 1B).^[^ ^14^ ^]^ Receiver‐operating characteristic (ROC) curve analysis revealed high area under the curve (AUC) values for *SV2B* when comparing TFE3‐RCC with ARTs, KIRP, and KIRC (Figure 1C). Using public data, we found that *SV2B* showed diagnostic efficacy comparable to that of *TRIM63* but significantly superior to that of *GPNMB* in distinguishing TFE3‐RCC from KIRC and KIRP (Figure S1, Supporting Information). *SV2B* mRNA expression was also associated with high ROC AUC values in our cohort (Figure 1C). Western blot and IHC analyses further supported the elevated expression of SV2B in TFE3‐RCC tissues compared to ARTs, KIRC, and KIRP (Figure 1D,E; Figure S2, Supporting Information). In contrast, SV2B expression was negative in ARTs and KIRP samples, with only marginal expression in KIRC samples (Figure 1E; Figure S2, Supporting Information). These findings suggested that SV2B may serve as an effective diagnostic marker for TFE3‐RCC.

### 2.2 SV2B is a Direct Target of TFE3

**Figure 2:**
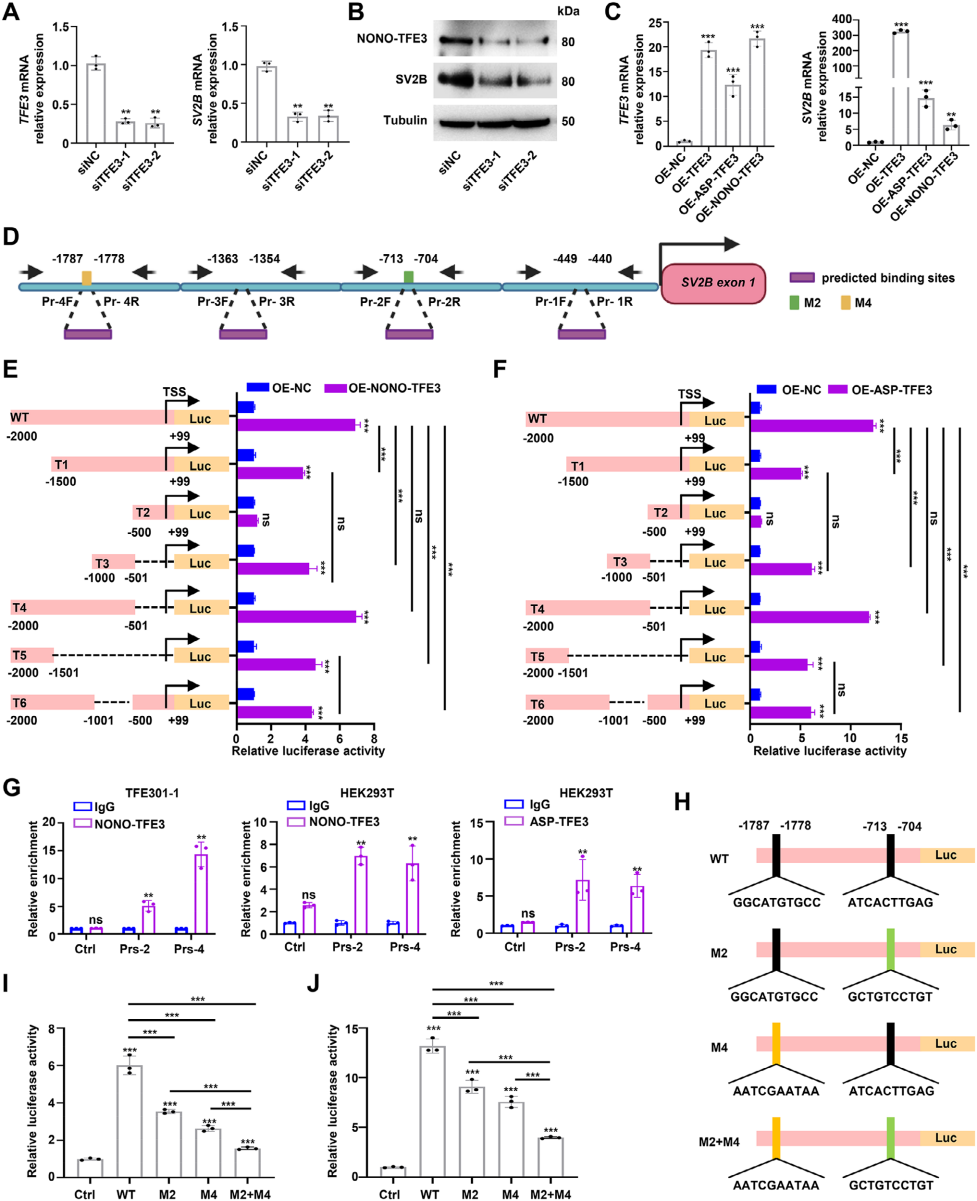
SV2B is a direct target of TFE3. A,B) SV2B and TFE3 expression in TFE301‐1 cells with TFE3 knockdown. C) *SV2B* and *TFE3* expression in HEK293T cells with TFE3 and TFE3 fusion proteins overexpression. D) Schematic depiction of DNA sequences upstream of *SV2B* transcriptional start site (TSS), with four predicted binding sites for TFE3. Primer 2 was designed for TFE3 binding site ‐713 to ‐704 and Primer 4 was designed for TFE3 binding site ‐1787 to ‐1778. Schematic representation created with Biorender.com. E,F) HEK293T cells were cotransfected with various truncated segments of the *SV2B* promoter‐expressing plasmid and TFE3 fusion gene‐overexpressing plasmid or a negative control plasmid. The transcriptional activity of the TFE3 fusion proteins on *SV2B* was assessed using a double luciferase reporter assay. G) ChIP‐qPCR was performed to confirm binding of TFE3 fusion proteins to sites ‐713 to ‐704, and ‐1787 to ‐1778 upstream of *SV2B* TSS in TFE301‐1 and HEK293T cells. H) Schematic diagram of *SV2B* promoter mutants. I,J) The transcriptional activity of the NONO‐TFE3 and ASP‐TFE3 fusion proteins on *SV2B* was assessed using a double luciferase reporter assay, respectively. These experiments were replicated three times. Data are presented as the mean ± SD. ns, not significant; **p < 0.01; ***p < 0.001.

Knockdown of *TFE3* led to a decrease in SV2B expression in TFE301‐1 cells, whereas overexpression of *TFE3* or a *TFE3* fusion gene in HEK293T cells enhanced SV2B expression (**Figure** **2**A–C). The JASPAR database predicted four potential TFE3 binding regions in the *SV2B* promoter region, including ‐449 to ‐440, ‐713 to ‐704, ‐1363 to ‐1354, and ‐1787 to ‐1778 upstream of the *SV2B* transcription start site (TSS) (Figure 2D). Luciferase assays confirmed that NONO‐TFE3 and ASPSCR1‐TFE3 enhanced SV2B transcription by binding to the regions ‐713 to ‐704 and ‐1787 to ‐1778 upstream of the *SV2B* TSS (Figure 2E,F).

Chromatin immunoprecipitation (ChIP)‐qPCR analysis in TFE01‐1 cells demonstrated significant enrichment of the NONO‐TFE3 fusion protein at two regions upstream of the *SV2B* TSS: ‐713 to ‐704 and ‐1787 to ‐1778 (Figure 2G). This binding profile mirrors that observed in HEK293T cells exogenously expressing either NONO‐TFE3 or ASPSCR1‐TFE3 fusion proteins (Figure 2G). Enrichment peaks were not detected in the regions ‐449 to ‐440 and ‐1363 to ‐1354 upstream of the *SV2B* TSS (Figure S3A, Supporting Information). While mutations in the regions ‐713 to ‐704 and ‐1787 to ‐1778 slightly reduced the binding ability of NONO‐TFE3 or ASPSCR1‐TFE3 to the *SV2B* promoter, simultaneous mutation of both sites strongly, but not completely, reduced the binding ability of the TFE3 fusion proteins to the *SV2B* promoter (Figure 2H–J). Additionally, luciferase assays demonstrated that the transcriptional activation of *SV2B* by the TFE3 fusion proteins requires the bHLH domain and its specific binding to the *SV2B* promoter regions (Figure S3B,C, Supporting Information). These results indicated that *SV2B* is a direct target gene of TFE3 fusion proteins and is transcriptionally regulated by them.

### 2.3 SV2B is Involved in Promoting the Progression of TFE3‐RCC

**Figure 3:**
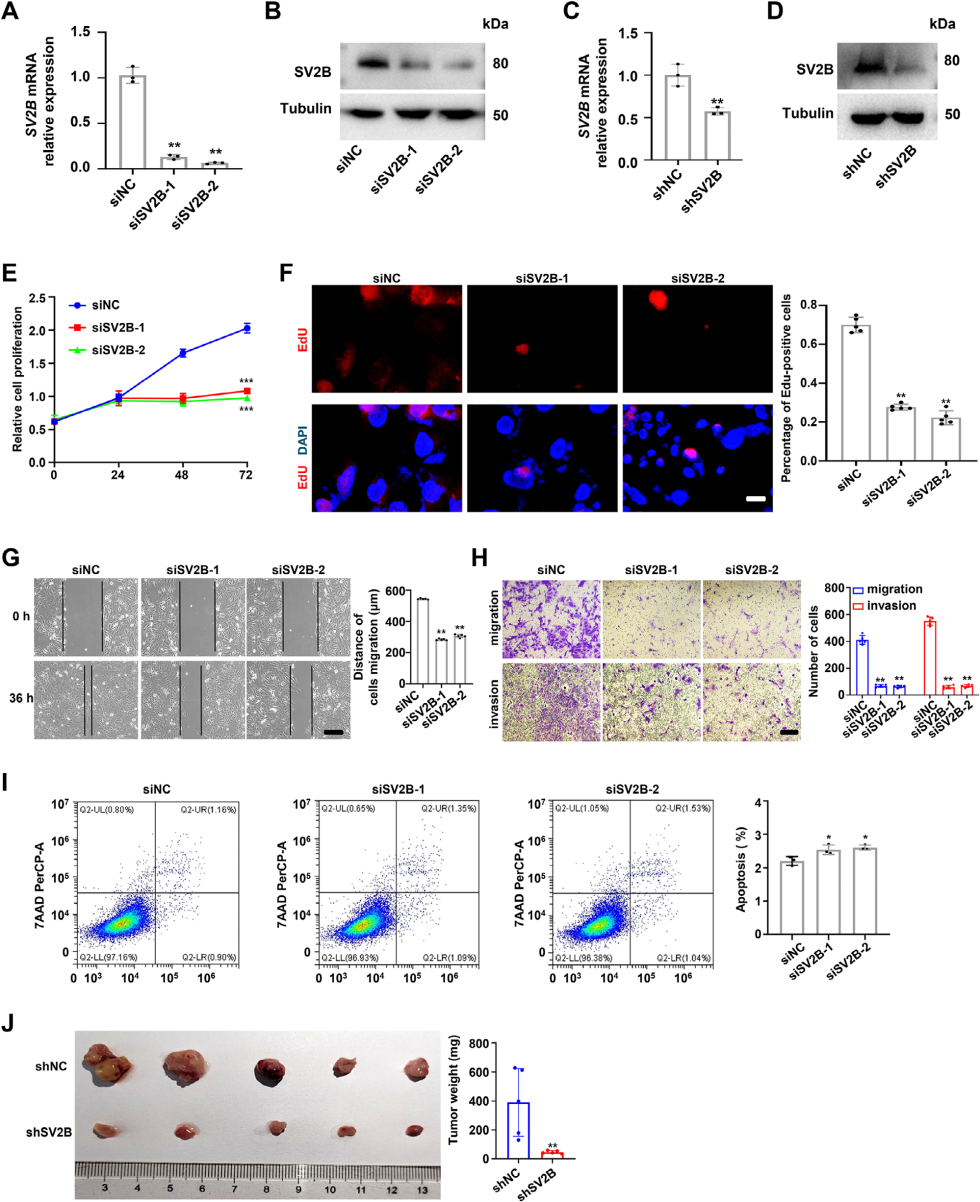
SV2B is involved in promoting the progression of TFE3‐RCC. A,B) SV2B knockdown with siRNAs in TFE301‐1 cells was verified by qPCR and western blot, respectively. C,D) SV2B knockdown with shRNAs in TFE301‐1 cells was verified by qPCR and western blot, respectively. E,F) SV2B promoted the proliferation of TFE301‐1 cells. Scale bars = 30 µm. G,H) SV2B promoted the migration and invasion of TFE301‐1 cells. Scale bars =100 µm. I) SV2B slightly inhibited the apoptosis of TFE301‐1 cells. J) SV2B knockdown inhibited the proliferation of TFE301‐1 cells in vivo. These experiments were replicated three times. Data are presented as the mean ± SD. **p < 0.01; ***p < 0.001.

Knockdown of *SV2B* in TFE301‐1 cells resulted in reduced proliferation, migration, and invasion, as well as slightly increased apoptosis (**Figure** **3**A,B,E–J). Overexpression of NONO‐TFE3 or ASP‐TFE3 fusion proteins promoted proliferation in HEK293T cells, which was reverted upon *SV2B* knockdown (Figure S4, Supporting Information). In a subcutaneous xenograft model, mice injected with *SV2B*‐knockdown TFE301‐1 cells developed lighter and smaller tumors than control mice (Figure 3C,D,J). Overall, these findings indicated that SV2B plays a crucial role in promoting the progression of TFE3‐RCC both in vitro and in vivo.

### 2.4 SV2B Suppresses RELA/NFKB1 Ubiquitination, Leading to NF‐κB Pathway Activation

**Figure 4:**
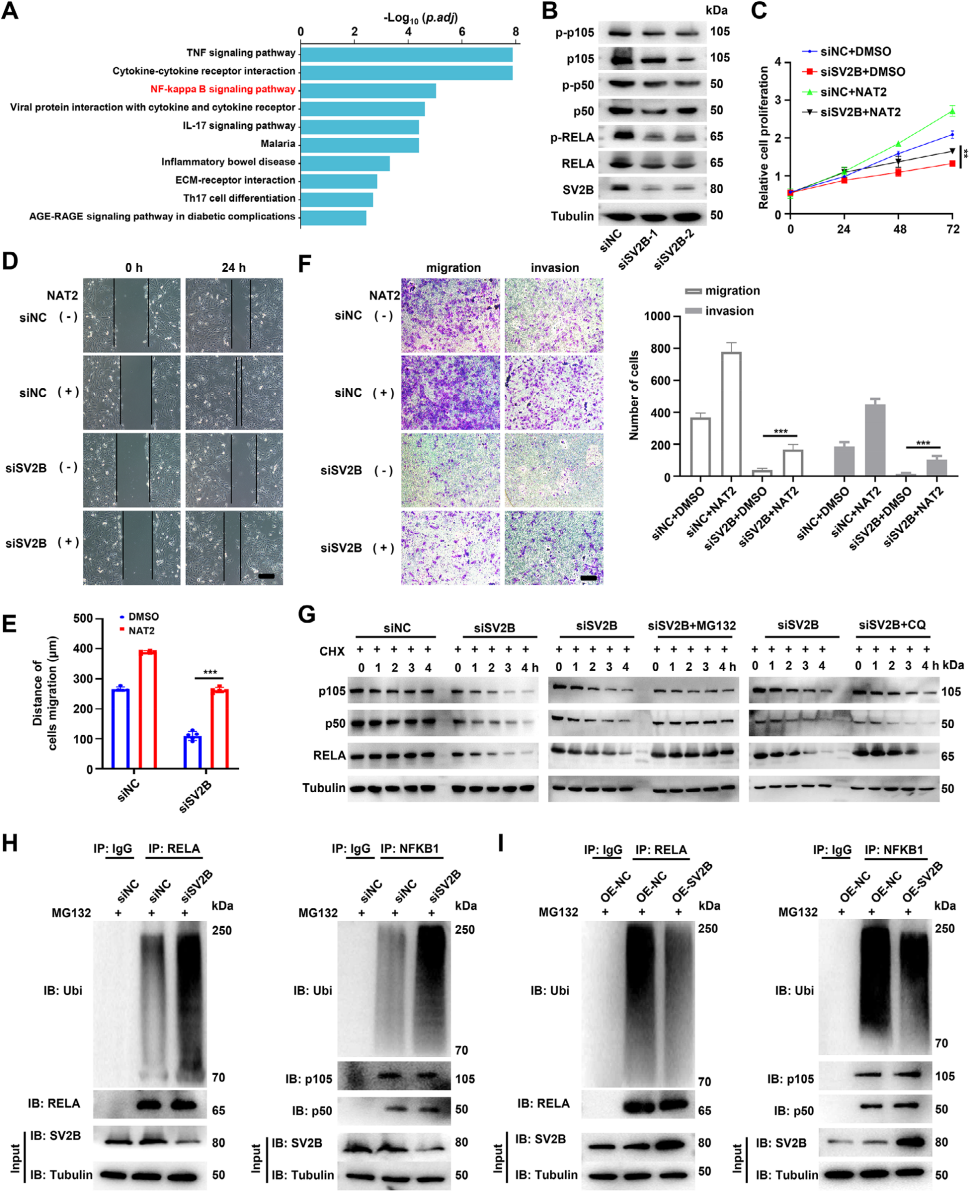
SV2B suppresses the ubiquitination of RELA/NFKB1, leading to the activation of the NF‐κB signaling pathway. A) Differential expressed genes were analyzed by KEGG pathway enrichment analysis and shown by histogram chart. B) RELA and NFKB1 protein expression were down‐regulated in SV2B knockdown cell. C–F) Treatment with an NF‐κB activator partially rescued the phenotypes induced by SV2B knockdown. G) The half‐life of RELA and NFKB1 proteins decreased after SV2B knockdown. RELA and NFKB1 protein degradation were rescued by MG132 treatment, but not by CQ. H) Knockdown of SV2B significantly enhanced the ubiquitination of RELA and NFKB1. I) Overexpression of SV2B significantly suppressed the ubiquitination of RELA and NFKB1. These experiments were replicated three times. Data are presented as the mean ± SD. Scale bars = 100 µm; **p < 0.01; ***p < 0.001; NAT2, NF‐κB activator 2.

The molecular mechanisms of SV2B in TFE3‐RCC were investigated using RNA sequencing of wild‐type and *SV2B*‐knockdown TFE301‐1 cells. Analysis of the differentially expressed genes in *SV2B*‐knockdown TFE301‐1 cells (Figure S5A, Supporting Information) revealed that the NF‐κB signaling pathway was downregulated (**Figure** **4**A). Downstream targets of NF‐κB signaling, such as BCL2A1, CCL22, CCL4, CSF1, HMOX1, and MMP9, were also downregulated (Figure S5B,C, Supporting Information). In contrast, NF‐κB signaling was activated in HEK293T cells overexpressing SV2B (Figure S5D, Supporting Information). To validate the relationship between SV2B and the NF‐κB pathway, we overexpressed *NONO‐TFE3* or *ASP‐TFE3* while knocking down *SV2B* in HEK293T cells (Figure S5E, Supporting Information). As expected, *SV2B* knockdown rescued the upregulation of NF‐κB signaling targets caused by overexpression of the *TFE3* fusion genes in HEK293T cells (Figure S5F, Supporting Information). Importantly, treatment with NF‐κB activator 2 (an NF‐κB activator) partially rescued the proliferation, migration, and invasion induced by *SV2B* knockdown in TFE301‐1 cells (Figure 4C–F).

Notably, the levels of RELA and NFKB1 proteins were down‐regulated in SV2B knockdown cells (Figure 4B), while their mRNA levels remained unchanged (Figure S5B, Supporting Information). This suggested that SV2B knockdown may affect the degradation rate of RELA and NFKB1 proteins. Protein degradation mainly occurs through the proteasomal or lysosomal pathways. Treatment with cycloheximide (CHX) in SV2B knockdown cells led to a rapid decrease in RELA and NFKB1 protein half‐life, which could be rescued by MG132 but not by chloroquine (CQ) (Figure 4G), indicating that RELA and NFKB1 are degraded through the proteasome pathway. Knockdown of *SV2B* increased the ubiquitination levels of RELA and NFKB1, whereas overexpression of *SV2B* suppressed their ubiquitination (Figure 4H,I). We found no direct interactions between SV2B and RELA/NFKB1 (Figure S6, Supporting Information), suggesting that SV2B indirectly suppresses the ubiquitination of RELA/NFKB1.

### 2.5 SV2B Interacts with HERC2 to Inhibit RELA/NFKB1 Degradation via the Ubiquitin‐Proteasome Pathway

**Figure 5:**
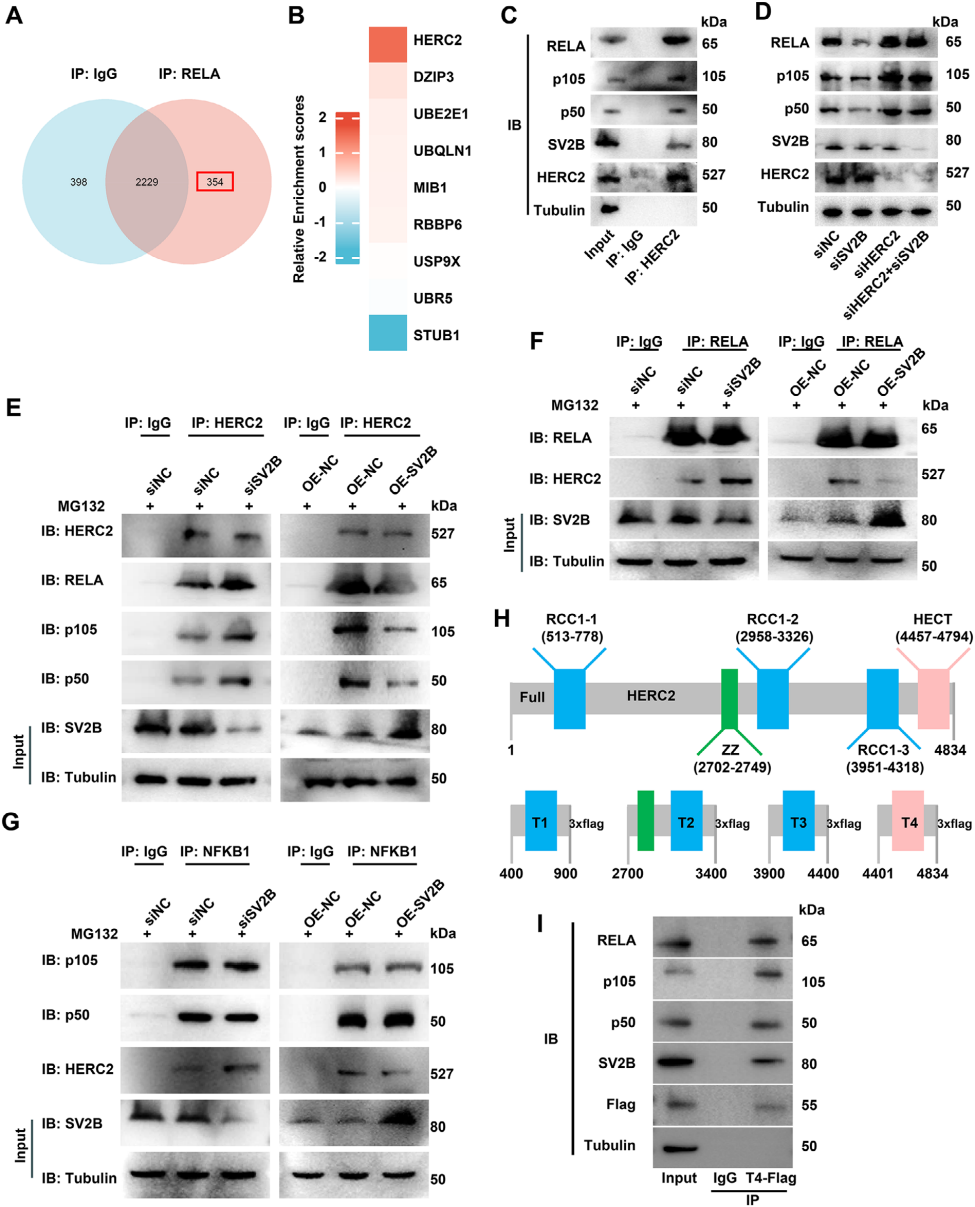
SV2B interacts with HERC2, inhibiting the degradation of RELA/NFKB1 through the ubiquitin‐proteasome pathway. A) RELA‐bound proteins were analyzed by mass spectroscopy and compared with those pulled down in an IgG control. B) HERC2 was the top one E3 ubiquitin ligase identified by IP‐MS analysis. C) HERC2 interacted with SV2B and RELA/NFKB1. D) HERC2 knockdown rescued the RELA/NFKB1 protein expression induced by knockdown of SV2B in TFE301‐1 cells. E–G) The interaction between HERC2 and RELA/NFKB1was attenuated once SV2B was overexpressed and vice versa. H) Schematic depiction of human HERC2 protein, showing the location of conserved domains (residue numbers are bracketed), including an HECT domain, a ZZ‑type zinc finger (ZZ), and clusters of RCC1 repeats. HERC2 truncation constructs were generated: T1 (aa 400–900), T2 (aa 2700–3400), T3 (aa 3900–4400), and T4 (aa 4401–4834). I) T4 truncation variant interacted with SV2B and RELA/NFKB1. These experiments were replicated three times.

Immunoprecipitation‐mass spectrometry (IP‐MS) analysis was used to identify key components of the ubiquitin‐proteasome pathway involved in the degradation of RELA proteins in TFE301‐1 cells (**Figure** **5**A). Nine enzymes associated with ubiquitination, including HERC2, DZIP3, UBE2E1, UBQLN1, MIB1, RBBP6, USP9X, UBR5, and STUB1, were identified as potential RELA interactors (Figure 5B). Among the nine ubiquitin‐related proteins examined, HERC2 exhibited the highest enrichment abundance in the IP‐MS analysis, and the interaction between HERC2 and RELA/NFKB1 was confirmed using immunoblotting (Figure 5B,C). Knockdown of HERC2 rescued the suppressive effects on proliferation, migration, and invasion caused by SV2B knockdown in TFE301‐1 cells (Figure S7, Supporting Information). HERC2 expression was significantly negatively correlated with NF‐κB enrichment scores (R = ‐0.604, *p* = 0.025), whereas the expression levels of the other eight enzymes were not significantly correlated with NF‐κB enrichment (Figure S8, Supporting Information). Co‐IP assays to determine whether DZIP3 and UBE2E1, the next two most abundant proteins identified in IP‐MS analysis, also participate in SV2B‐NF‐κB signaling demonstrated that neither of these proteins interacts with SV2B (Figure S9A,B, Supporting Information).

HERC2 induced the ubiquitination of RELA and NFKB1, whereas *HERC2* knockdown significantly inhibited their ubiquitination (Figure S9C,D, Supporting Information). Both SV2B and RELA/NFKB1 were found to interact with HERC2, whereas SV2B did not interact with RELA/NFKB1 (Figure 5C; Figure S6, Supporting Information). When SV2B was overexpressed, the interaction between HERC2 and RELA/NFKB1 was weakened, and vice versa (Figure 5E–G), suggesting that SV2B competes with HERC2 for binding to RELA/NFKB1. To identify the specific domains of HERC2 responsible for binding SV2B and RELA/NFKB1, we constructed various HERC2 truncation variants and performed Co‐IP assays (Figure 5H). Only the T4 truncation variant bound to both SV2B and RELA/NFKB1, indicating that the HECT domain of HERC2 is a critical binding site for these interactions (Figure 5I; Figure S9E, Supporting Information).

In conclusion, the interaction between the HECT domain of HERC2 and RELA/NFKB1 is hindered by SV2B, which binds to the HECT domain of HERC2 to prevent the degradation of RELA/NFKB1.

### 2.6 Padsevonil or Withaferin A Effectively Target TFE3‐RCC Progression

**Figure 6:**
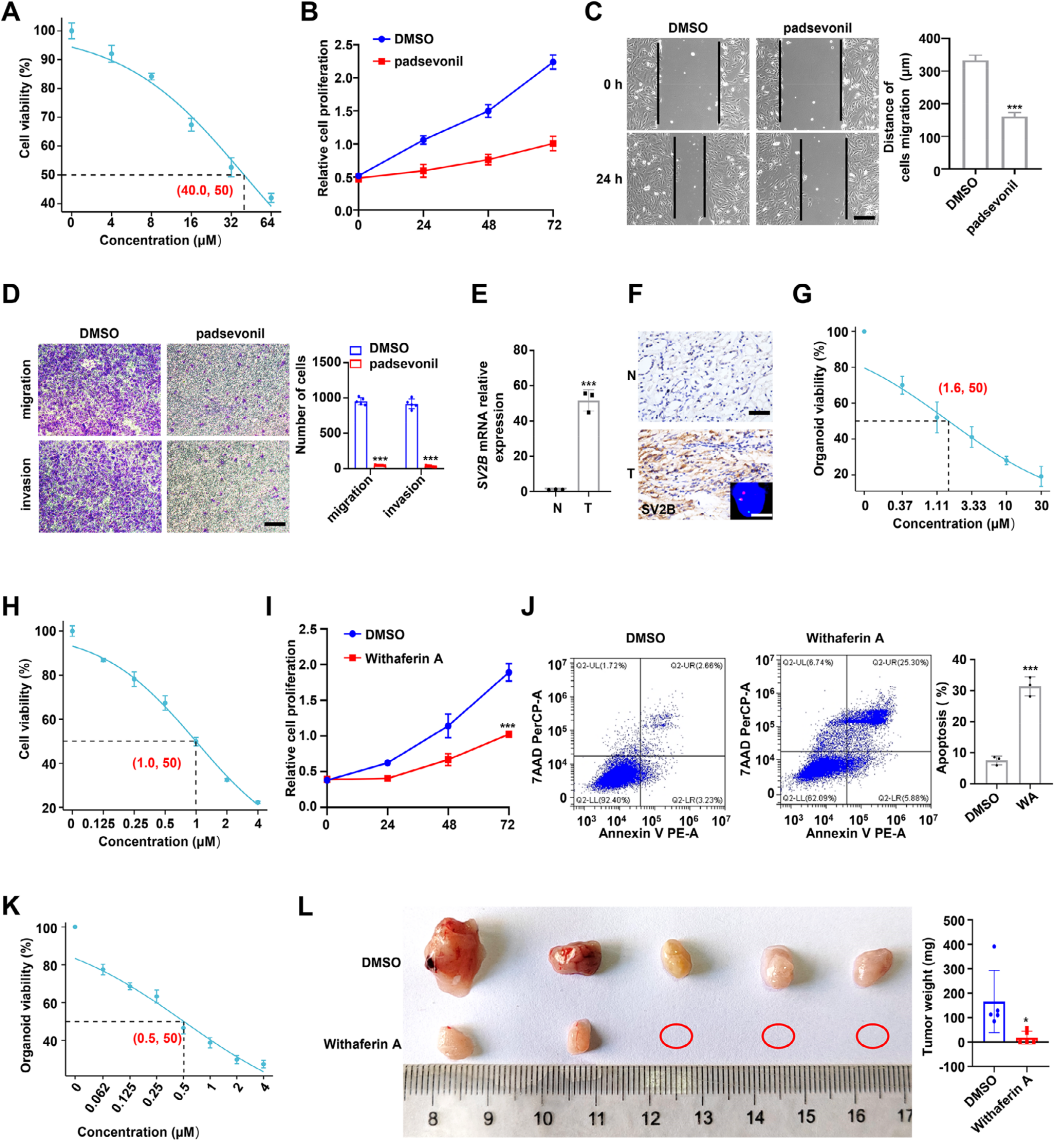
Padsevonil or Withaferin A effectively targets TFE3‐RCC progression. A) The IC_50_ value of padsevonil against TFE301‐1 cells was about 40 µm. B–D) Padsevonil at 50% IC_50_ concentration inhibited the proliferation, migration, and invasion of TFE301‐1 cells. Scale bars =100 µm. E,F) SV2B expression in a pair of fresh TFE3‐RCC tumor tissue and matched adjacent tissue. Scale bars =30 µm. G) The IC_50_ value of padsevonil against TFE3‐RCC organoid was about 1.6 µm. H) The IC_50_ value of Withaferin A against TFE301‐1 cells was about 1.0 µm. I,J) Withaferin A at 50% IC_50_ concentration inhibited the proliferation and induced apoptosis of TFE301‐1 cells. K) The IC_50_ value of Withaferin A against TFE3‐RCC organoid was about 0.5 µm. L) Withaferin A inhibited the proliferation of TFE301‐1 cells in vivo. These experiments were replicated three times. Data are presented as the mean ± SD. *p < 0.05; ***p < 0.001; N, Normal tissues; T, Tumor tissues.

Padsevonil, a new antiepileptic drug developed by UCB Pharma, is designed to specifically target SV2 isoforms. In a phase III clinical trial (NCT03739840) in patients with drug‐resistant epilepsy, padsevonil was generally well tolerated and safe. We found that padsevonil suppressed the growth, migration, and invasion of TFE301‐1 cells, whereas it did not affect cell death and the cell cycle in these cells (**Figure** **6**A–D; Figure S10A–C, Supporting Information). The IC_50_ value of padsevonil in TFE301‐1 cells was approximately 40 µM (Figure 6A). Padsevonil did not significantly inhibit the growth of A498 KIRC cells, OSRC2 RCC cells, or normal tubular epithelial HK2 cells (Figure S11, Supporting Information), which expressed relatively low levels of *SV2B*. (Figure S11C, Supporting Information). In addition, *SV2B* expression was substantially higher than that of *SV2A* and *SV2C* in TFE3‐RCC tissues (Figure S11D, Supporting Information), suggesting that padsevonil inhibited tumor progression in TFE3‐RCC primarily by targeting SV2B. Consistent herewith, TFE301‐1 cells treated with padsevonil exhibited reduced levels of NF‐κB pathway activation (Figure S10D,E, Supporting Information). TFE3‐RCC organoids were successfully established and passaged to investigate the anti‐tumor effect of padsevonil (Figure 6E,F; Figure S10F–H, Supporting Information). Organoid morphology and proliferation capacity were compared between padsevonil‐ and non‐treated organoids. Padsevonil inhibited TFE3‐RCC organoid proliferation, with an IC_50_ value of 1.6 µM (Figure 6G). *SV2B* mRNA expression was significantly higher in TFE3‐RCC organoids than in TFE301‐1 cells, which may explain why the organoids were more sensitive to padsevonil (Figure S10I, Supporting Information). Moreover, 3D tumor organoid models are known to be more sensitive to drugs than cell cultures.^[^ ^27^ ^]^ Importantly, *SV2B* expression was low or undetectable in healthy major human tissues and organs (Figure S3D, Supporting Information). Hence, padsevonil has potential as a treatment for TFE3‐RCC.

Withaferin A effectively prevents NF‐κB activation by blocking the TNF‐induced activation of IκB kinase beta. In a phase II clinical trial for recurrent ovarian cancer, Withaferin A was safe and well‐tolerated after oral administration, with no adverse reactions observed at a dose of 500 mg.^[^ ^28^ ^]^ Withaferin A inhibited the progression of TFE301‐1 cells (Figure 6H–J; Figure S12A,B, Supporting Information), but not HKC normal tubular epithelial cells or KIRC or OSRC2 cells (Figure S12C–F, Supporting Information). To explore the anti‐tumor effect of Withaferin A in vivo, TFE301‐1 cells were injected subcutaneously into the right flank of NOD scid gamma (NSG) mice to establish a traditional xenograft nude mouse model. Mock‐treated control animals developed large tumors (Figure 6L). In contrast, three out of five animals treated with Withaferin A did not develop macroscopic tumors, while the remaining two developed significantly smaller tumors (Figure 6L). To further investigate its anti‐tumor treatment effect, Withaferin A was tested in TFE3‐RCC organoids. Similar to padsevonil, Withaferin A effectively inhibited organoid proliferation (Figure 6K), indicating its potential as a promising agent for treating TFE3‐RCC.

### 2.7 TFE3‐RCC Activates the TFE3‐SV2B‐NF‐κB Axis

**Figure 7:**
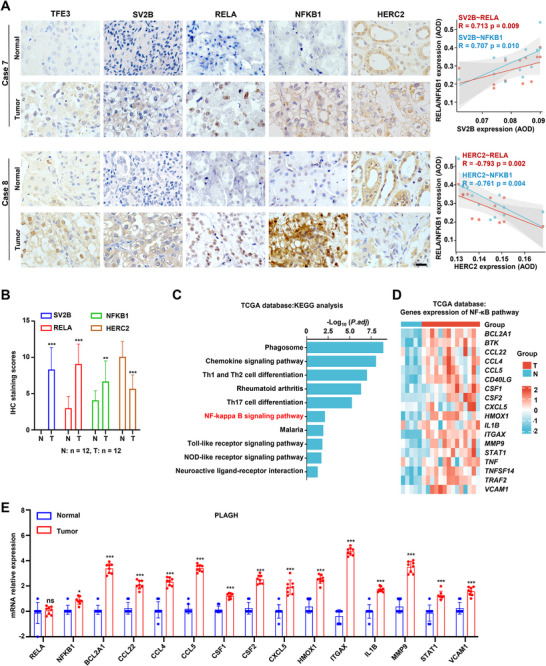
TFE3‐RCC activates the TFE3‐SV2B‐NF‐κB axis. A) TFE3, SV2B, RELA, NFKB1, and HERC2 protein expression in TFE3‐RCC tissues and ARTs in our cohort. Correlation analysis was performed with Spearman's correlation test. B) The results of IHC were scored, and SV2B, RELA, and NFKB1 expression in TFE3‐RCC were higher. C) KEGG pathway enrichment analysis using the TCGA database indicated NF‐κB pathway activation in TFE3‐RCC. D) Expression of RELA downstream target genes was investigated using TCGA database. E) Expression of RELA downstream target genes was investigated in our cohort. These experiments were replicated three times. Data are presented as the mean ± SD. Scale bars =30 µm; *p < 0.05; **p < 0.01; ***p < 0.001; N, Normal tissues; T, Tumor tissues; AOD, The average optical density.

The clinical significance of the TFE3‐SV2B‐NF‐κB axis in TFE3‐RCC was assessed by analyzing TFE3, SV2B, HERC2, RELA, and NFKB1 protein levels in our cohort. The results revealed a positive correlation between SV2B and RELA/NFKB1 expression, whereas HERC2 expression was negatively correlated with that of RELA/NFKB1 (**Figure** **7**A). SV2B protein levels were significantly higher in TFE3‐RCC tissues than in ARTs (Figure 7A,B). Nuclear accumulation of TFE3 was enhanced in TFE3‐RCC tissues (Figure 7A).

RELA and NFKB1 expression levels were significantly higher in TFE3‐RCC than in ARTs, with both proteins primarily localizing in cell nuclei in TFE3‐RCC tissues (Figure 7A,B). Kyoto Encyclopedia of Genes and Genomes (KEGG) pathway analysis using The Cancer Genome Atlas (TCGA) database revealed that NF‐κB pathway was activated in TFE3‐RCC (Figure 7C). A heatmap generated using TCGA data showed elevated levels of NF‐κB downstream targets in TFE3‐RCC versus ARTs (Figure 7D). In our cohort, the expression of key downstream RELA targets, including BCL2A1, CCL22, CCL4, CSF1, HMOX1, and MMP9, was elevated in TFE3‐RCC tissues (Figure 7D,E). These findings validated the activation of the TFE3‐SV2B‐NF‐κB axis in TFE3‐RCC tissues.

## 3 Discussion

Our study revealed that *SV2B* is a direct target gene of TFE3 fusion proteins and may serve as a useful biomarker for TFE3‐RCC. This discovery presents a new clinical diagnostic approach for TFE3‐RCC. Additionally, SV2B plays a role in promoting TFE3‐RCC progression via the TFE3‐SV2B‐NF‐κB axis, shedding light on the mechanisms underlying TFE3‐RCC progression. Selective targeting of SV2B or the NF‐κB pathway inhibited TFE3‐RCC progression, offering two potential clinical treatment options for this disease.

SV2B was identified as a novel indicator that can effectively differentiate TFE3‐RCC from KIRC and KIRP. TFE3, MLANA, HMB45, and CTSK are not sufficiently reliable for distinguishing TFE3‐RCC from KIRC and KIRP.^[^ ^8^, ^9^ ^]^ While strong positive nuclear staining of TFE3 in TFE3‐RCC is typically associated with TFE3 fusion protein expression, some KIRC or KIRP cases stain positive for TFE3 because of autophagy activation, leading to potential false‐positive diagnoses. Additionally, the level of *CTSK* transcriptional activation by TFE3 may vary among TFE3 fusion proteins.^[^ ^7^, ^29^ ^]^ We previously reported NMRK2 as a diagnostic biomarker for TFE3‐RCC,^[^ ^14^ ^]^ and the present study uncovered SV2B as another diagnostic marker for TFE3‐RCC. Both SV2B and NMRK2 stained positive in all TFE3‐RCC tissues, offering new possibilities for the clinical use of diagnostic markers.

SV2B is a direct target gene of TFE3 fusion proteins. A recent study showed that all TFE3 fusion isoforms retain exons 7–10 of *TFE3*, which comprise the bHLH and leucine zipper domains, but only a subset retain the transcription activation domain.^[^ ^30^ ^]^ Unlike TFE3, NONO‐TFE3 is a weak transcriptional activator that lacks a strong transcriptional activation domain.^[^ ^30^ ^]^ This may explain why the NONO‐TFE3 fusion upregulated SV2B expression less potently than TFE3 did. In addition, different TFE3 fusions exhibit significant variation in expression abundance, nuclear localization efficiency, and DNA‐binding capacity,^[^ ^31^ ^]^ accounting for the differential SV2B expression observed in TFE3‐RCC patients carrying different *TFE3* fusion genes.

We uncovered a new role for the SV2B protein in promoting tumor growth. SV2B enhances TFE3‐RCC progression by activating the NF‐κB pathway, which is a crucial pathway that promotes or inhibits tumor progression depending on the cell type.^[^ ^32^, ^33^ ^]^ Our study showed that NF‐κB is activated and plays a cancer‐promoting role in TFE3‐RCC. RELA and NFKB1 protein stability is regulated by the ubiquitin‐proteasome pathway.^[^ ^34^, ^35^ ^]^ E3 ubiquitin ligases such as PDLIM2, PPARγ, and HERC3 target RELA for degradation.^[^ ^36^, ^37^, ^38^ ^]^ We identified HERC2 as a novel E3 ubiquitin ligase that interacts with RELA/NFKB1, leading to their degradation. Interestingly, SV2B competes with HERC2 for binding, preventing HERC2 from interacting with RELA/NFKB1 and thus inhibiting their degradation. Similarly, IκBα has been found to prevent the interaction between HERC3 and RELA.^[^ ^38^ ^]^ In addition to the NF‐κB pathway, SV2B may affect other major signaling pathways, as suggested by our transcriptome sequencing results, which remain to be validated.

We found that targeting SV2B or the NF‐κB pathway effectively inhibited the progression of TFE301‐1 cells and TFE3‐RCC organoids. Current treatments for advanced TFE3‐RCC, such as tyrosine kinase inhibitors, mTOR inhibitors, and immune checkpoint inhibitors, are not consistently effective in all patients.^[^ ^2^, ^15^, ^16^, ^17^, ^18^ ^]^ This may be because TFE3‐RCC is considered an “immunologically cold tumor,” and not all cases involve EGFR or mTOR activation.^[^ ^30^ ^]^ SV2B is highly expressed in TFE3‐RCC and can promote its progression via the NF‐κB pathway. Therefore, targeting SV2B or the NF‐κB pathway may be promising therapeutic approaches for TFE3‐RCC. Our study showed that padsevonil and Withaferin A had significant inhibitory effects on TFE3‐RCC. Recent studies have demonstrated that these drugs are safe and well‐tolerated after oral administration, highlighting their clinical translational potential for the treatment of TFE3‐RCC.^[^ ^28^, ^39^ ^]^ Notably, SV2B is expressed at low levels in other major organs and tissues. Hence, targeting SV2B may result in fewer toxic side effects than targeting the NF‐κB pathway.^[^ ^40^ ^]^ In conclusion, we suggest targeting SV2B or the NF‐κB pathway as novel therapeutic strategies for TFE3‐RCC.

The current study had some limitations. The number of TFE3‐RCC cases was small, and the sample size needs to be expanded for further verification and analysis. Further, we used only male mice, not female mice in the in vivo experiments; therefore, it is unknown whether the findings are relevant for female mice. In future studies, we plan to include female mouse xenograft models as well.

## 4 Experimental Section

### Patients and Clinical Materials

The clinicopathological data of the patients with TFE3‐RCC, KIRC, and KIRP included in this study have been previously reported.^[^ ^14^ ^]^ In the present study, 12 TFE3‐RCC tissues, 12 TFE3‐RCC adjacent renal tissues (ARTs), 40 KIRP tissues, and 40 KIRC tissues were analyzed. The study was approved by the Ethics Committee of the Chinese People's Liberation Army General Hospital (approval No.: S2013‐065‐01), and informed consent was obtained from all the patients.

### Public Data Analysis

TCGA‐KIRC and TCGA‐KIRP datasets were obtained from the Genomics Data Commons portal of the National Cancer Institute (https://portal.gdc.cancer.gov/) and comprised 14 TFE3‐RCC tissues, five ARTs, 280 KIRP tissues, and 532 KIRC tissues. Additionally, the GSE167573^[^ ^30^ ^]^ dataset, comprising 63 TFE3‐RCC tissues and 14 ARTs, was acquired from the Gene Expression Omnibus (https://www.ncbi.nlm.nih.gov/geo/). ROC curves were generated using established methodologies.^[^ ^14^ ^]^

### Quantitative Reverse Transcription (q)PCR

*SV2B* mRNA expression levels in TFE3‐RCC, KIRP, and KIRC tissues and ARTs were quantified using qPCR with the forward primer GAGGAGAACACCTCAGTTGGC and the reverse primer CAGAGCACAGACGATGACAAAC. qPCRs were run using established protocols, as previously reported.^[^ ^41^ ^]^

### Western Blotting

Western blotting was performed using established methodologies, as previously described.^[^ ^42^ ^]^ Antibodies against SV2B (orb357011; Biorbyt, Cambridge, UK), SV2B (sc‐166004; Santa Cruz Biotechnology [SCBT], Santa Cruz, CA, USA), TFE3 (HPA023881; Sigma‐Aldrich, Darmstadt, Germany), RELA (8242; Cell Signaling Technology [CST], Danvers, MA, USA), RELA (sc‐8008; SCBT), phospho‐RELA (3033; CST), NFKB1 (14220‐1‐AP; Proteintech, Rosemont, IL, USA), NFKB1 (sc‐166588; SCBT), phospho‐NFKB1 (sc‐271908; SCBT), HERC2 (27459‐1‐AP; Proteintech), HERC2 (sc‐515891; SCBT), ubiquitin (80992‐1‐RR; Proteintech), ubiquitin (sc‐271289; SCBT), DYKDDDDK‐tagged polyclonal antibody (20543‐1‐AP; Proteintech), UBE2E1 (sc‐136113; SCBT), DZIP3 (sc‐514725; SCBT), and β‐tubulin (BE0025; EASYBIO, Beijing, PR China) were used.

### IHC Staining

IHC staining was used to examine SV2B, RELA, NFKB1, HERC2, and TFE3 protein levels in TFE3‐RCC tissues and ARTs, SV2B levels in KIRC and KIRP tissues, and PAX8, TFE3, SV2B levels in TFE3‐RCC organoids. Antibodies against SV2B (14624‐1‐AP; Proteintech), TFE3 (HPA023881; Sigma‐Aldrich), RELA (8242; CST), NFKB1 (14220‐1‐AP; Proteintech), HERC2 (27459‐1‐AP; Proteintech), and PAX8 (10336‐1‐AP; Proteintech) were used. The integrated optical density and the area of the region of interest were measured using ImageJ. The average optical density corresponds to the “Mean” value provided by ImageJ.^[^ ^43^, ^44^ ^]^ Standard protocols were used, as described previously.^[^ ^45^ ^]^

### Cell Culture

HEK293T, HKC, HK2, A498, and OSRC2 cells were obtained from the National Platform of Experimental Cell Resources for Sci‐Tech (Beijing, China) in September 2015 and authenticated in January 2022.^[^ ^46^ ^]^ The TFE301‐1 cell line was immortalized from TFE3‐RCC primary cells, as described in the previous publication.^[^ ^14^ ^]^ HEK293T cells were cultured and maintained in Dulbecco's modified Eagle's medium (DMEM; Gibco, Grand Island, NY, USA). A498 cells were cultured and maintained in MEM (Gibco). HKC, HK2, and TFE301‐1 cells were cultured and maintained in DMEM/F12 (Gibco). OSRC2 cells were cultured and maintained in RPMI 1640 (Gibco). The medium was supplemented with 10% fetal bovine serum (Evergreen, Hangzhou, China) and 1% penicillin–streptomycin. All cell lines were passaged less than 20 times and incubated at 37 °C in 5% CO_2_ under mycoplasma‐free conditions.

### Plasmid Construction and Transfection

The complete coding sequences of TFE3, NONO‐TFE3, and ASPSCR1‐TFE3 were cloned into the PCDH‐CMV‐EF1A‐copGFP‐T2A‐PURO‐3×Flag vector. The coding sequences of NONO‐TFE3 lacking the bHLH domain (NONO‐TFE3‐bHLH) or ASPSCR1‐TFE3 lacking the bHLH domain (ASP‐TFE3‐bHLH) were cloned into the PCDH‐CMV‐EF1A‐copGFP‐T2A‐PURO‐3×Flag vector. HEK293T cells were transfected with PCDH‐CMV‐EF1A‐copGFP‐T2A‐PURO‐3×Flag‐TFE3, PCDH‐CMV‐EF1A‐copGFP‐T2A‐PURO‐3×Flag‐NONO‐TFE3, or PCDH‐CMV‐EF1A‐copGFP‐T2A‐PURO‐3×Flag‐ASPSCR1‐TFE3. HERC2 truncation constructs T1 (aa 400–900), T2 (aa 2700–3400), T3 (aa 3900–4400), and T4 (aa 4401–4834) were cloned into the pcDNA3.1 (+)‐3×Flag vector. Small interfering (si)RNAs targeting *TFE3*, *SV2B*, *RELA*, *NFKB1*, and *HERC2*, and negative control siRNA were procured from GenePharma (Suzhou, China). Short hairpin (sh)RNAs targeting *SV2B* (pCLenti‐U6‐shSV2B‐CMV‐EGFP‐F2A‐BSR‐WPRE) and a negative control shRNA (pCLenti‐U6‐shNC‐CMV‐EGFP‐F2A‐BSR‐WPRE) were acquired from OBIO (Shanghai, China). Transfections were performed using the jetPRIME transfection reagent (101000046; Sartorius, Illkirch‐Graffenstaden, France) per the manufacturer's instructions.

### 5‐Ethynyl‐2′‐Deoxyuridine (EdU) Staining

Edu staining assays were performed using standard techniques, as previously reported.^[^ ^42^ ^]^

### Cell Counting Kit‐8 (CCK8) Assay

Cell proliferation was assessed using the CCK8 assay (Cat: CK001, Lablead Biotech), according to the manufacturer's instructions.

### Wound‐Healing Assay

Wound‐healing assays were performed using standard techniques, as previously reported.^[^ ^42^ ^]^

### Migration and Invasion Assays

Migration and invasion assays were performed using standard techniques, as previously reported.^[^ ^42^ ^]^ Migration was monitored for 24 h, and invasion for 24 or 36 h.

### Flow Cytometry

TFE301‐1 cells were transfected with siRNA for 48 h or treated with Withaferin A or padsevonil for 24 h. Apoptosis and the cell cycle were analyzed using flow cytometry, as previously reported.^[^ ^14^ ^]^

### ChIP Assay

ChIP assays were performed using standard techniques, as previously reported.^[^ ^14^ ^]^ qPCR was performed on the purified DNA using the primer sets listed in Table S4 (Supporting Information).

### Luciferase Assay and Site‐Directed Mutagenesis

PsiCHECK‐2‐*SV2B* promoter luciferase constructs were developed by incorporating the *SV2B* promoter sequence into the psiCHECK‐2 luciferase reporter plasmid. The *SV2B* promoter was defined as the region spanning from –2000 to +100 base pairs relative to the TSS. Various truncated segments of the *SV2B* promoter, including T1 (+99 to –1500), T2 (+99 to –500), T3 (–501 to –1000), T4 (–501 to –2000), T5 (–1501 to –2000), and T6 (+99 to –500 and –1001 to –2000), were subcloned into the psiCHECK‐2 luciferase reporter plasmid. Mutations in the E‐BOX region of the *SV2B* promoter, where NONO‐TFE3 and ASP‐TFE3 bind, were introduced using *SV2B* E‐box mutant primers. Mutation sites were designated as M2 for –713 to –704, M4 for –1787 to –1778, and M2+M4 for mutations at both –713 to –704 and –1787 to –1778. HEK293T cells were seeded into 24‐well plates at a density of 5×10^4^ cells per well and incubated for 24 h. The promoter vectors were co‐transfected with NONO‐TFE3 or ASP‐TFE3 into the cells using jetPRIME transfection reagent. Luciferase reporter gene assays were conducted using the Dual‐Glo Luciferase assay system (E2920; Promega, Madison, WI, USA).

### RNA Sequencing

To investigate the molecular mechanisms underlying the oncogenic role of SV2B in TFE3‐RCC, total RNA was extracted from TFE301‐1 cells transfected or not with siRNA targeting *SV2B* for 72 h, using TRIzol. RNA was qualified using the 5300 Bioanalyzer and quantified using an ND‐2000 spectrophotometer. RNA sequencing and data analysis were outsourced to Majorbio (Shanghai Majorbio Bio‐Pharm Technology, Shanghai, China).

### Subcutaneous Human Xenograft Model

Male NSG mice aged 4 to 6 weeks were procured from GemPharmatech. Stable *SV2B* knockdown (shSV2B) and control (shNC) TFE301‐1 cells (5 × 10^6^/mouse) were injected subcutaneously into the right flank of the mice. Mice bearing TFE301‐1 tumors were euthanized 12 weeks post‐implantation. To assess the therapeutic efficacy of Withaferin A in TFE3‐RCC, TFE301‐1 cells (3 × 10^6^ /mouse) were similarly injected into the right flank of NSG mice. Upon reaching the target tumor volume (50–75 mm^3^), the mice were divided into two treatment groups: 1) vehicle control (10% DMSO and 90% glyceryl trioctanoate) and 2) Withaferin A at a dosage of 2 mg kg^−1^. Five animals were randomly assigned to each group. Both the vehicle control and Withaferin A were administered intraperitoneally every other day. Throughout the study, mice were closely monitored for general health, body weight changes, and tumor volume. After 15 days of treatment, the animals were sacrificed. The Animal Ethics Committee of the Chinese People's Liberation Army General Hospital approved the study (approval No.: S2013‐115‐01).

### IP‐MS Analysis

Antibodies against RELA (8242; CST), NFKB1 (14220‐1‐AP; Proteintech), and HERC2 (27459‐1‐AP; Proteintech), Rabbit IgG Isotype Control Recombinant Antibody (98136‐1‐RR; Proteintech), Mouse IgG (B900620, Proteintech), and DYKDDDDK tag Monoclonal antibody (66008‐4‐Ig; Proteintech) were used. IP analysis was conducted using standard protocols, as described previously.^[^ ^47^ ^]^ MS analysis was outsourced to Biotechnology Corp. (Beijing, China) and performed using the Thermo Scientific EASY‐nLC 1000 System and Thermo Q‐Exactive MS system.

### Construction of Primary TFE3‐RCC Organoids and Drug‐Susceptibility Testing

TFE3‐RCC tissues were dissected, enzymatically digested, and centrifuged. Cell pellets were resuspended in renal cancer organoid medium with equal‐volume Matrigel. Fifty microliter aliquots were transferred into 24‐well plates, solidified at 37 °C, and supplemented with 500 µL of medium containing 1× anti‐apoptotic factors. Organoid growth was monitored using light microscopy. For susceptibility testing, organoids were digested, mixed with 60% Matrigel, and seeded on IBAC S1 micro‐organ chips. Test groups received serially diluted (1:3) Withaferin A (up to 5 µm) or padsevonil (up to 30 µm). After incubation, the medium was refreshed, and ATP activity was measured.

### Statistical Analysis

All statistical analyses were conducted using GraphPad Prism version 8.0 (GraphPad, Inc., La Jolla, CA, USA) or R v4.2.1 (The R Foundation for Statistical Computing, Vienna, Austria).

Differential expression was analyzed using the “limma” package (v3.52.2) in R.^[^ ^48^ ^]^ Data visualization in dot plots, line plots, and histograms was accomplished using GraphPad Prism software (v8.0). ROC curves were generated and AUCs were calculated using the “pROC” package (v1.18.0) in R. Correlations in gene expression were assessed using the Spearman rank coefficient in the “ggplot2” package (v3.4.4) in R. Significance was set to *P* < 0.05.

## Conflict of Interest

The authors declare no conflict of interest.

## Author Contributions

H.Y.F., Z.X., and S.Q.Y. contributed equally to this work. X.B.L., X.Z., and X.M. conceived and designed the study. X.B.L. and H.Y.F. participated in the experiment design. H.Y.F., X.B.L., Z.X., and S.Q.Y. performed the experiments. Q.B.H., B.J.W., X.Z., and X.M. provided patient samples. J.X.L., S.Q.C., K.L., X.H., and L.Q.W. collected clinical data. H.Y.F., J.X.L., L.Q.W., X.H., and T.J.P. contributed to statistical analysis. H.Y.F. and X.B.L. wrote the manuscript with help from all authors. All authors read and approved the final version of the manuscript.

## Supporting information



Supporting Information

## Data Availability

The data that support the findings of this study are available from the corresponding author upon reasonable request.
