# Supplementary material for: SV2B Promotes the Progression of TFE3‐Rearranged Renal Cell Carcinoma by Interacting with HERC2 to Impede the Degradation of NF‐κB Subunits
Source: Adv Sci (Weinh). 2025 Nov 6;13(1):e09443. doi: 10.1002/advs.202509443 (PMC12767095; doi:10.1002/advs.202509443)
Supplement: Supplementary file 1 — Supporting Information [file ADVS-13-e09443-s001.docx]

**Supplemental Figures**


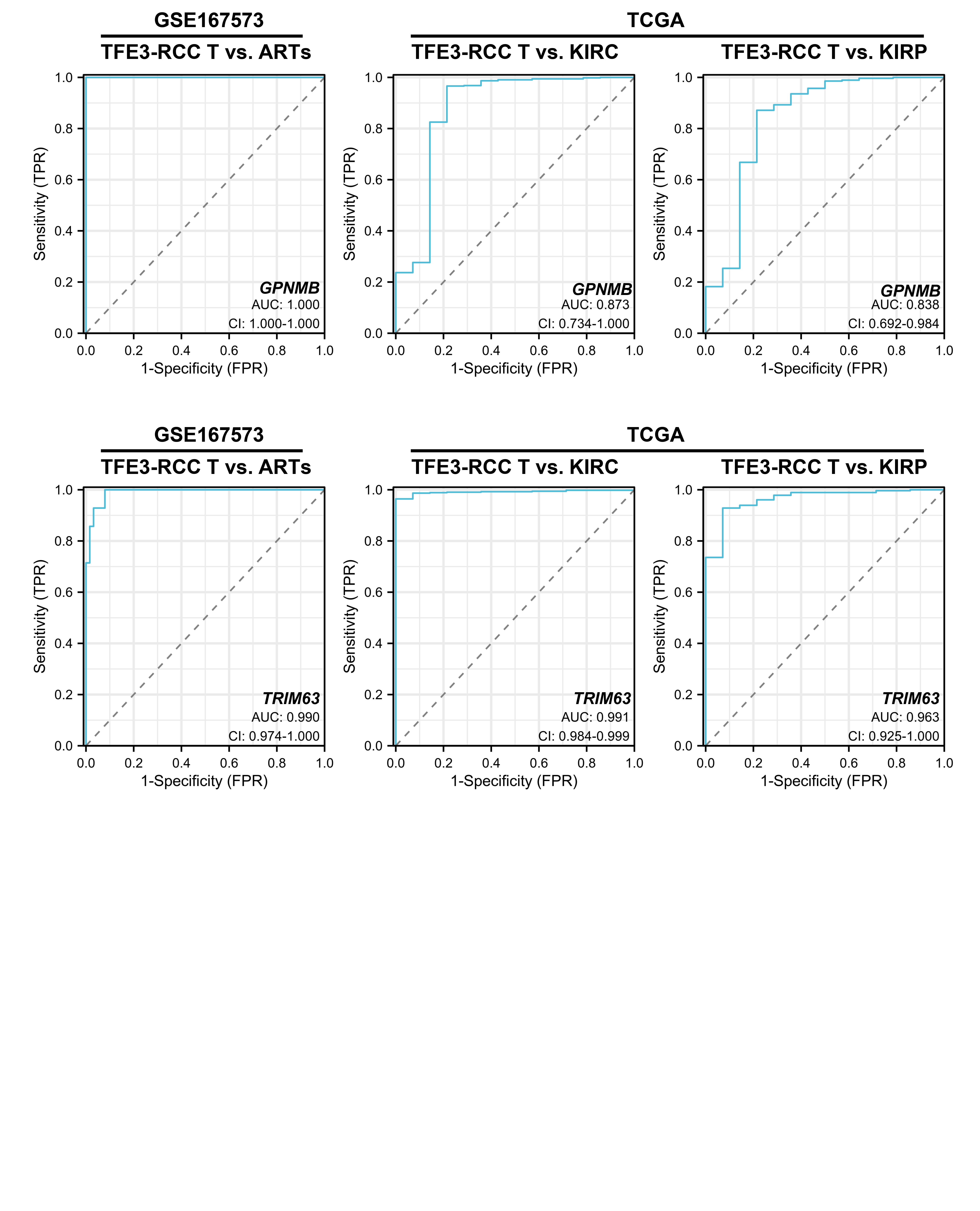


**Figure S1 Diagnostic performance of *GPNMB* and *TRIM63* in distinguishing TFE3-RCC from KIRC and KIRP.**

**A.** Sensitivity and specificity of *GPNMB* for distinguishing TFE3-RCC from ARTs, KIRP, and KIRC tissues using public databases. **B.** Sensitivity and specificity of *TRIM63* for distinguishing TFE3-RCC from ARTs, KIRP, and KIRC tissues using public databases.


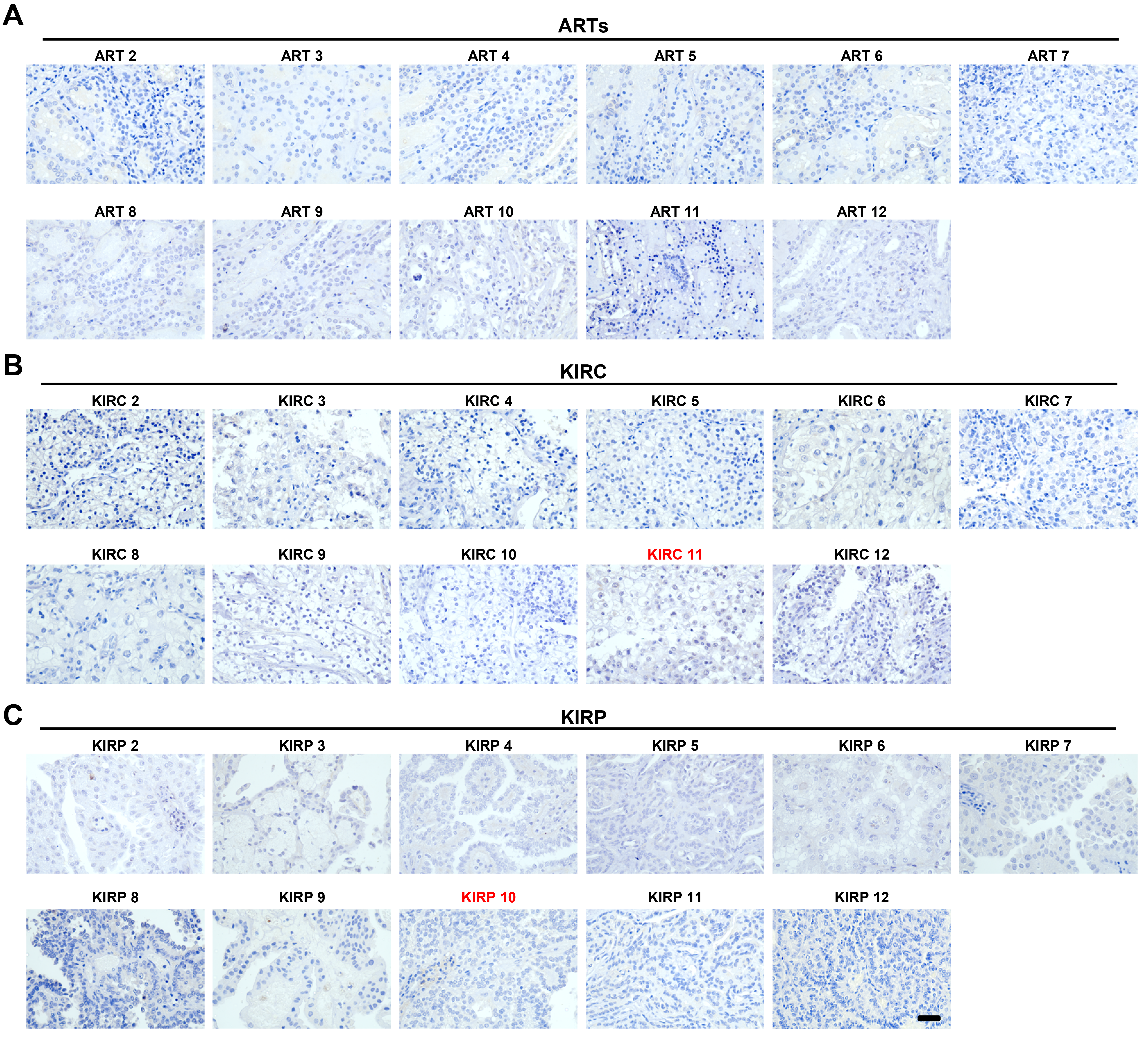


**Figure S2 SV2B is an effective diagnostic marker for distinguishing TFE3-RCC.**

**A**. IHC staining for SV2B was explored in ARTs in our cohort. **B**. IHC staining for SV2B was explored in KIRC in our cohort. **C**. IHC staining for SV2B was explored in KIRP in our cohort. These experiments were replicated three times. Scale bars = 100 μm.


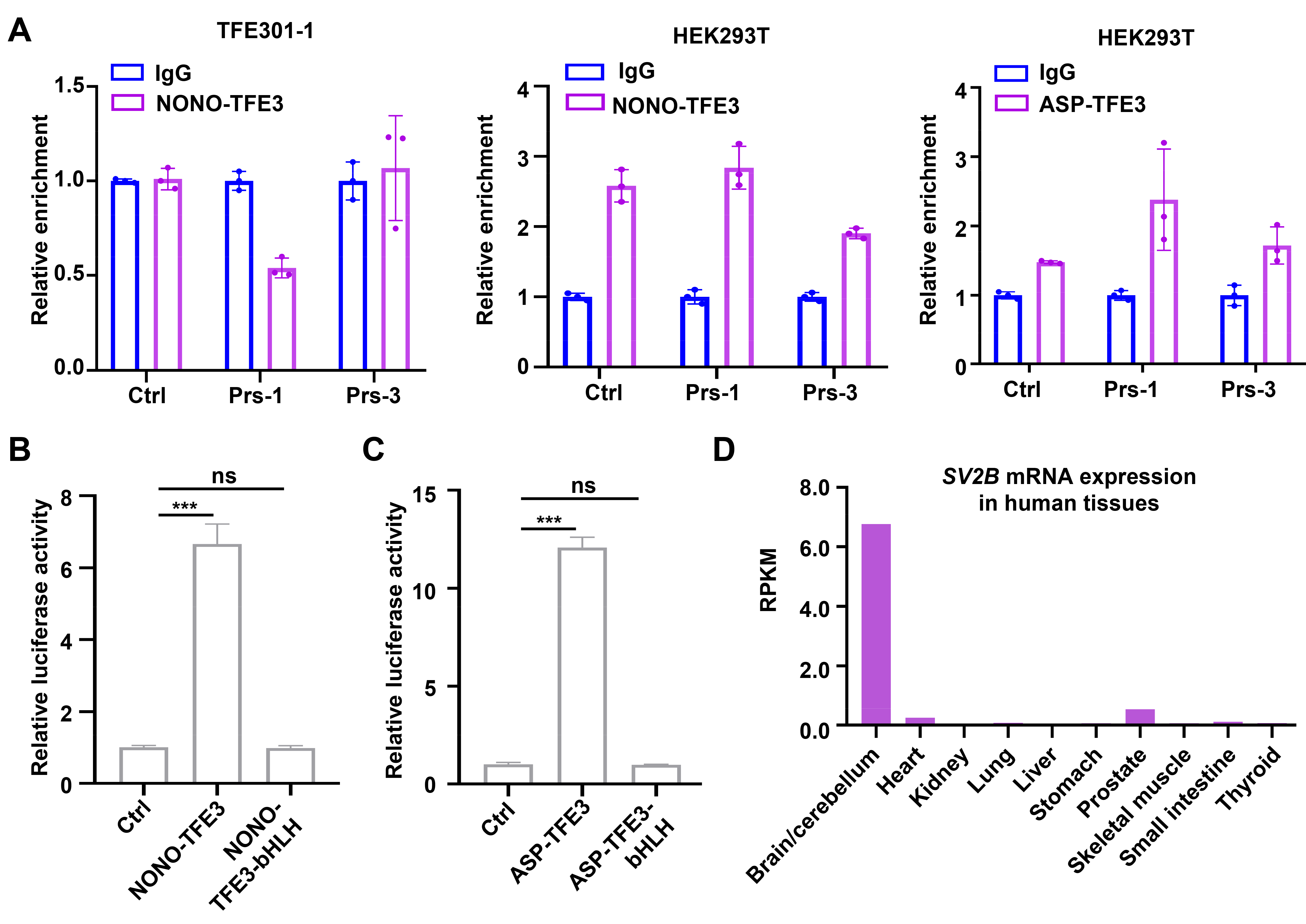


**Figure S3 *SV2B* is a direct target of TFE3**

**A**. ChIP-qPCR was performed to explore the binding of TFE3 fusion proteins to sites –449 to −440, and -1363 to -1354 upstream of *SV2B* TSS in TFE301-1 and HEK293T cells. **B.** The transcriptional activity of the NONO-TFE3 fusion protein lacking bHLH domain on *SV2B* was assessed using a double luciferase reporter assay. **C.** The transcriptional activity of the ASP-TFE3 fusion protein lacking bHLH domain on *SV2B* was assessed using a double luciferase reporter assay. **D.** *SV2B* mRNA expression in human tissues. Data sourced from the <https://www.ncbi.nlm.nih.gov/datasets/gene/9899/>. n = 1 per tissue type. These experiments were replicated three times. Data are presented as the mean ± SD. ns, not significant; ***, p < 0.001.


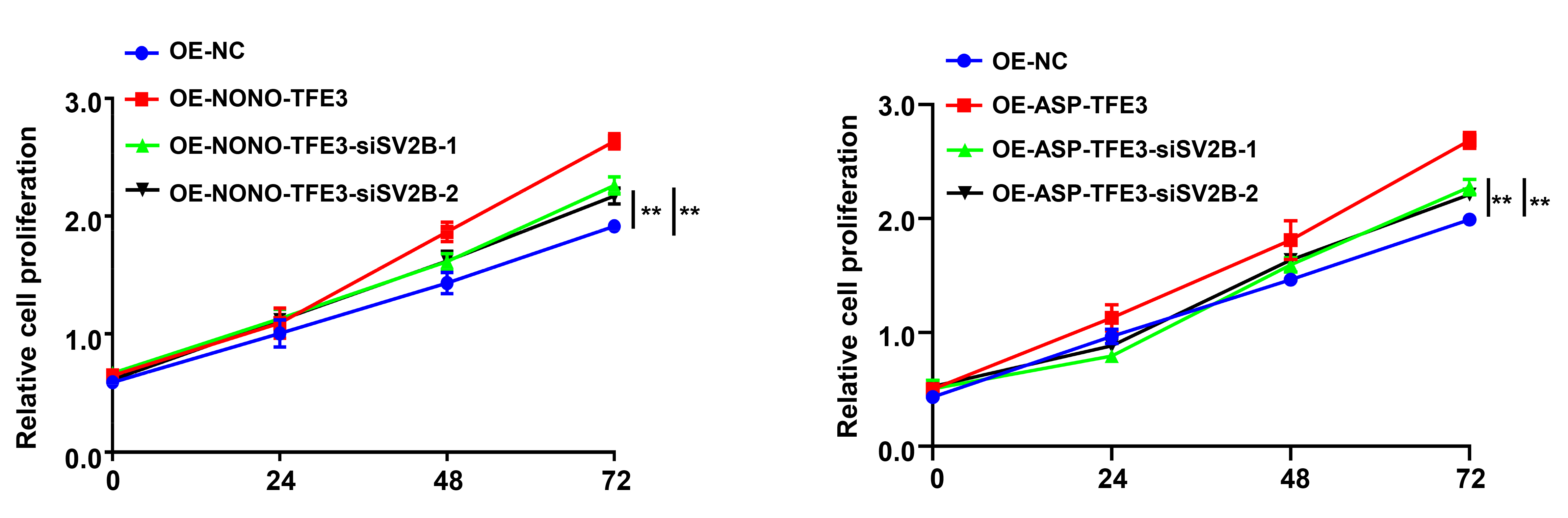


**Figure S4 SV2B mediates the function of TFE3 to promote the proliferation of TFE3-RCC.**

ASV2B knockdown rescued the proliferation induced by overexpression of TFE3 fusion proteins in HEK293T cells. **, p < 0.01.


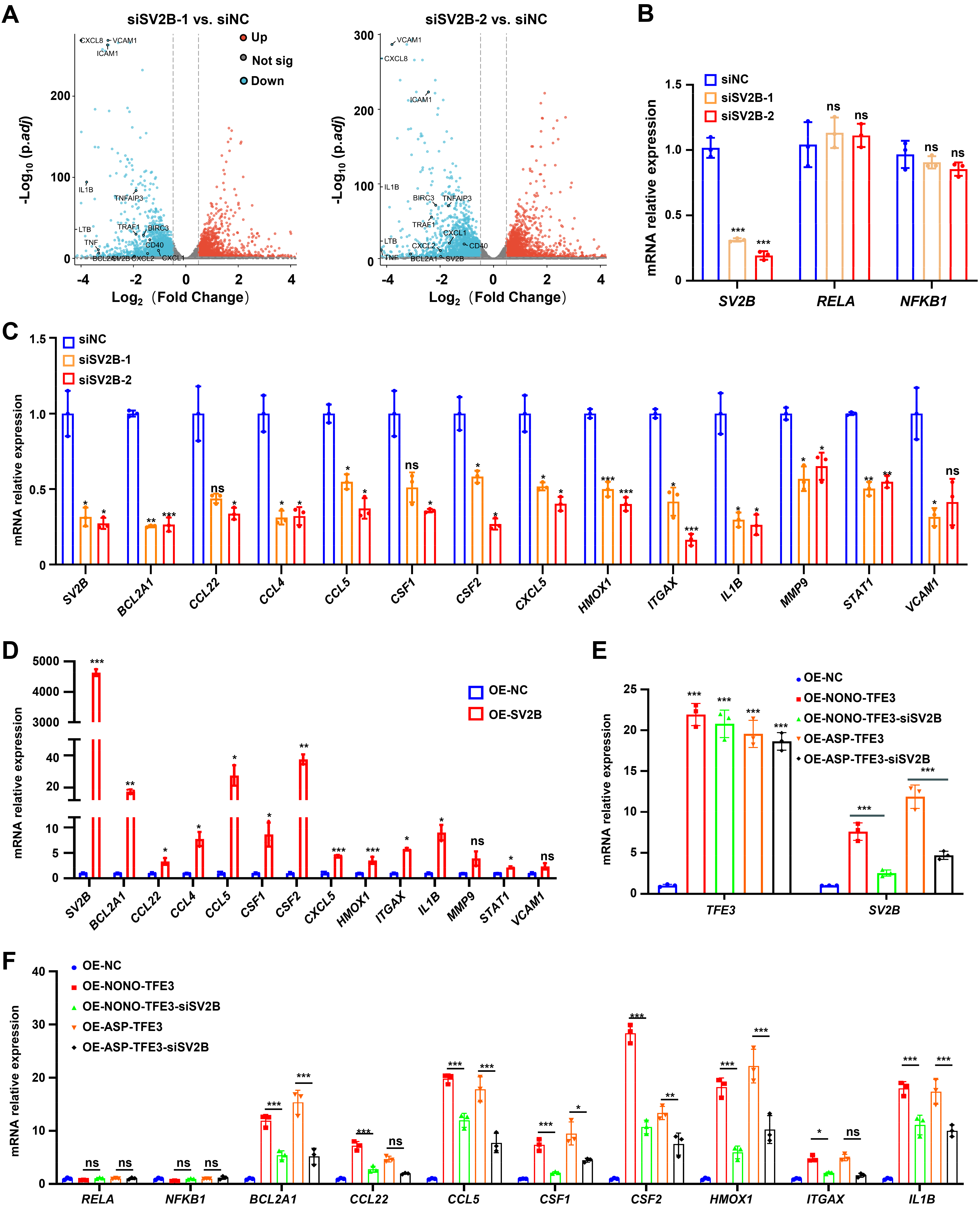
**Figure S5 SV2B activates the NF-κB signaling pathway in TFE3-RCC.**

**A**. Differentially expressed genes between siNC and siSV2B groups were shown in volcano plots. **B**. No significant down-regulation was detected at the mRNA level for *RELA* and *NFKB1* in SV2B knockdown cells. **C**. The downregulation of NF-κB signaling pathway downstream targets were confirmed in TFE301-1 cells. **D.** Exploring expression changes of NF-κB downstream target genes following SV2B overexpression in HEK293T cells. **E.** *TFE3* fusion genes overexpression and *SV2B* knockdown in HEK293T cells were verified by qPCR. **F**. Exploring expression changes of NF-κB downstream target genes following *TFE3* fusion genes overexpression and *SV2B* knockdown in HEK293T cells. These experiments were replicated three times. Data are presented as the mean ± SD. ns, not significant; *, p < 0.05; **, p < 0.01; ***, p < 0.001.


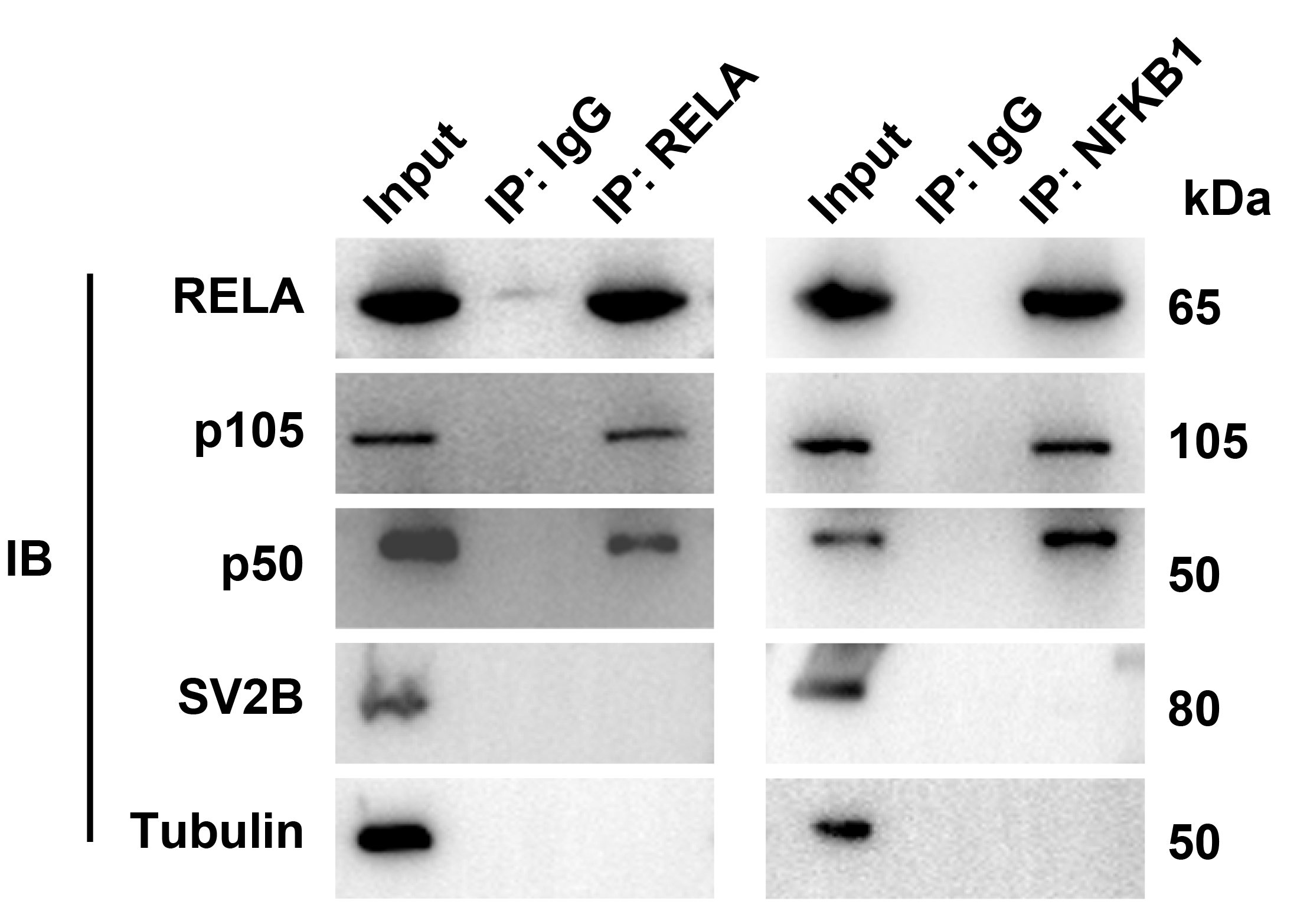


**Figure S6 No interactions between SV2B and RELA/NFKB1 were identified.**

Results of co-immunoprecipitation for RELA and NFKB1 with SV2B.


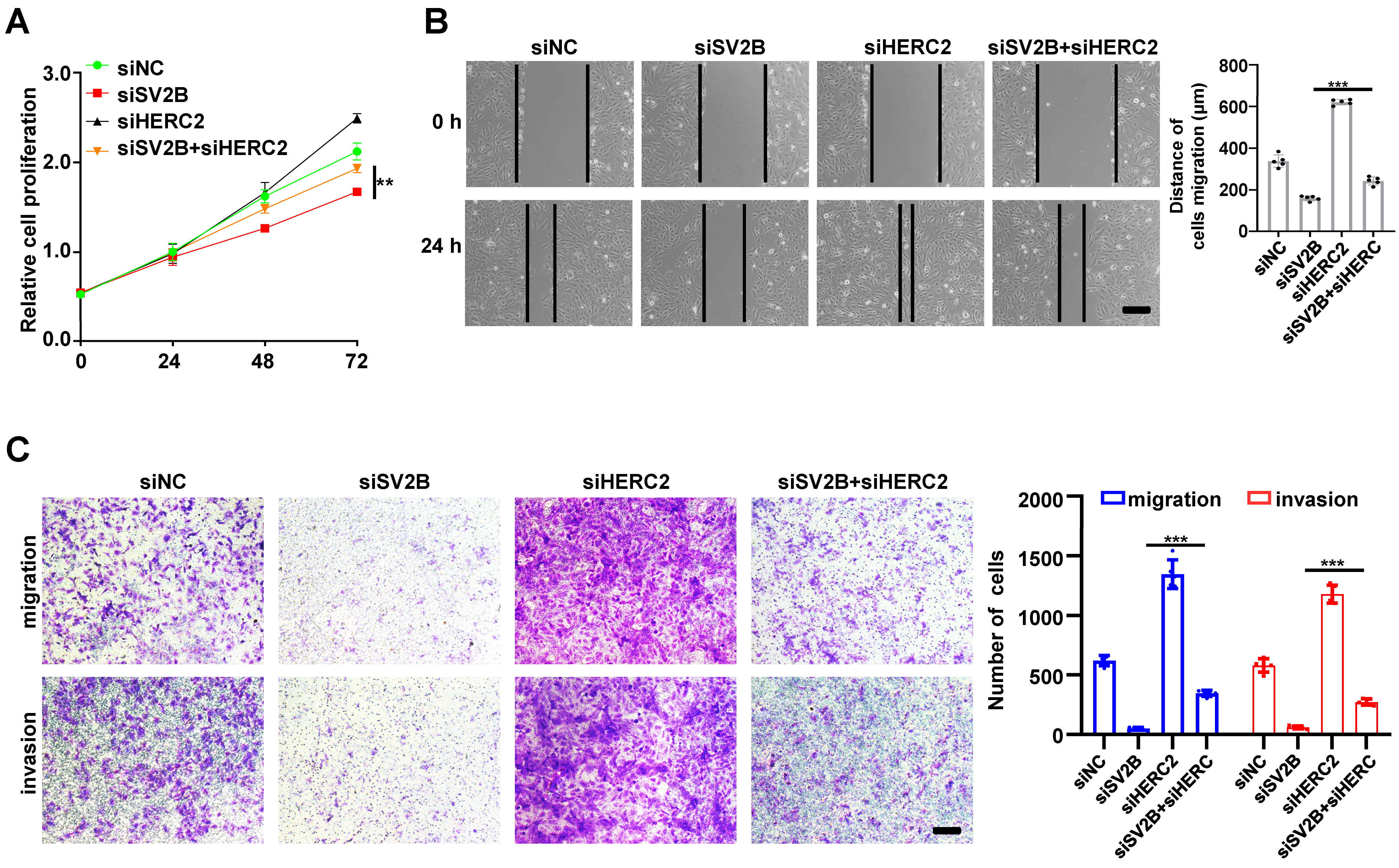


**Figure S7 HERC2 knockdown rescues the phenotypes induced by knockdown of SV2B in TFE301-1 cells.**

**A**. HERC2 knockdown rescued the suppression of proliferation caused by SV2B knockdown in TFE301-1 cells. **B**. HERC2 knockdown rescued the suppression of migration caused by SV2B knockdown in TFE301-1 cells. **C**. HERC2 knockdown rescued the suppression of migration and invasion caused by SV2B knockdown in TFE301-1 cells. These experiments were replicated three times. Data are presented as the mean ± SD. Scale bars = 100 μm; **, p < 0.01; ***, p < 0.001.


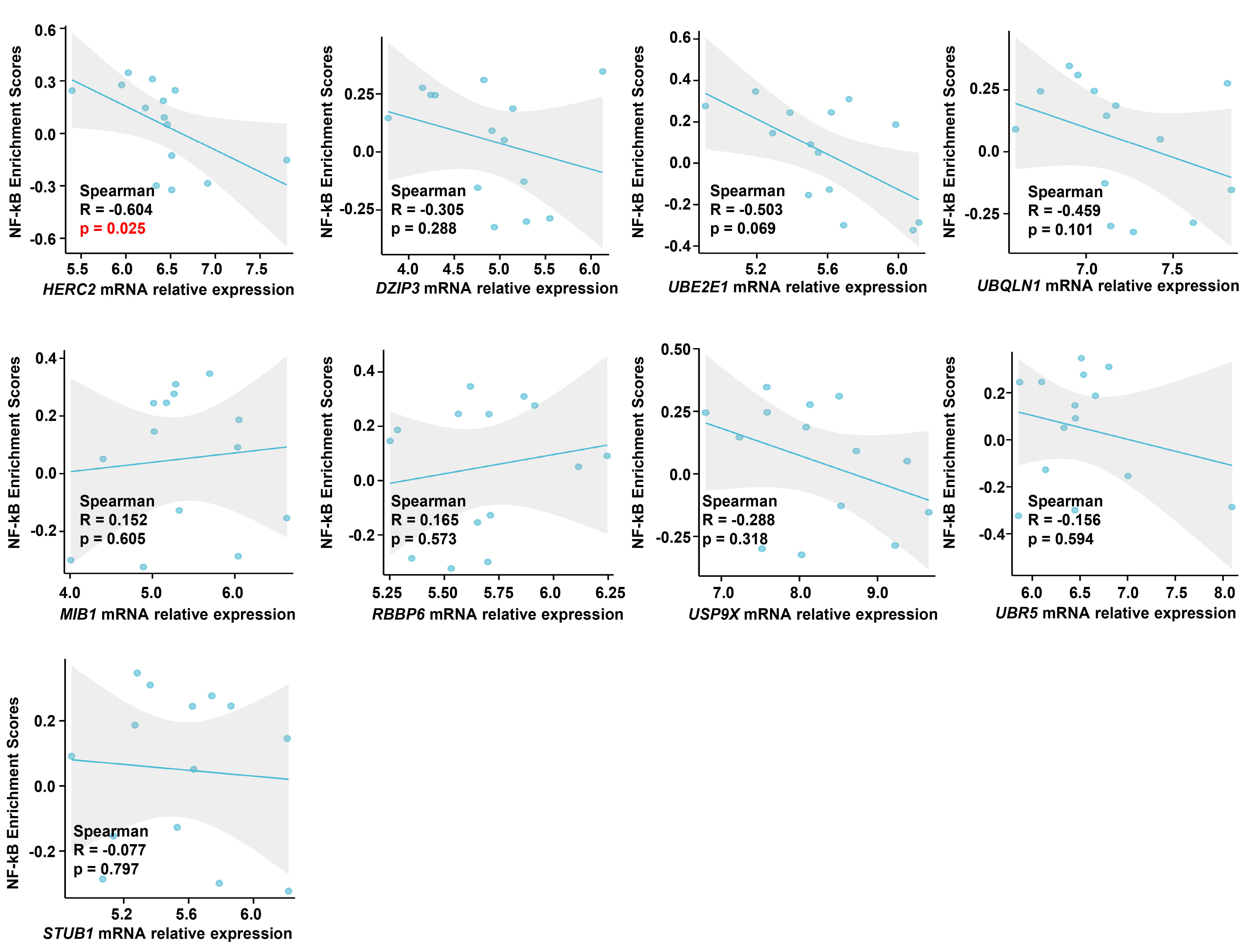


**Figure S8 *HERC2* expression negatively correlated with NF-κB enrichment scores.** A significant negative correlation between *HERC2* expression and NF-κB enrichment scores was identified using the TCGA database, whereas the correlations between the expression levels of the other eight enzymes and NF-κB enrichment scores were not statistically significant.


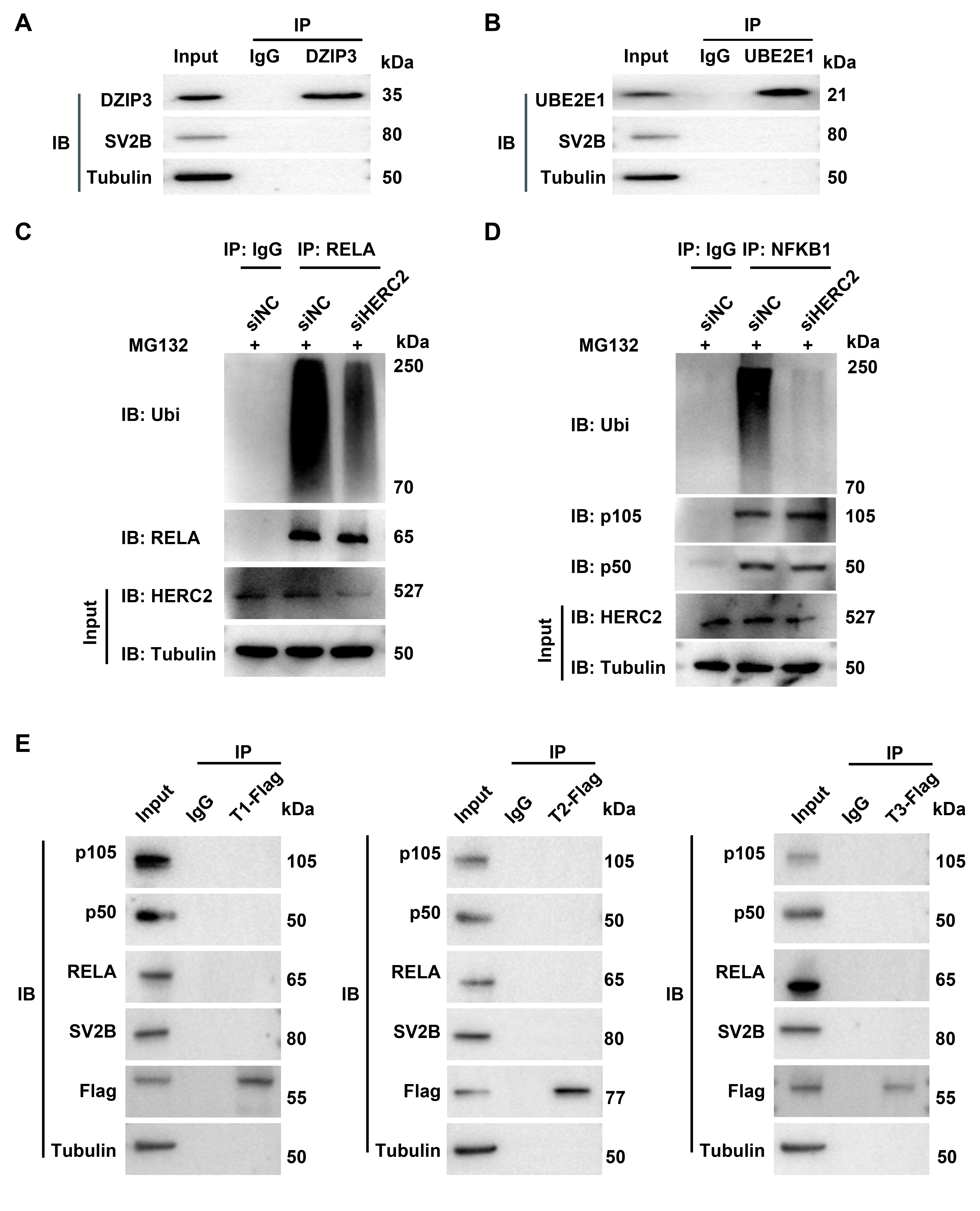


**Figure S9 SV2B interacts with HERC2, inhibiting the degradation of RELA/NFKB1 through the ubiquitin-proteasome pathway.**

**A.** Results of co-immunoprecipitation for DZIP3 with SV2B. **B.** Results of co-immunoprecipitation for UBE2E1 with SV2B. **C-D.** HERC2 knockdown inhibited the ubiquitination of RELA and NFKB1 significantly. **E**. Results of co-immunoprecipitation for HERC2 truncation variants with SV2B or RELA/NFKB1.


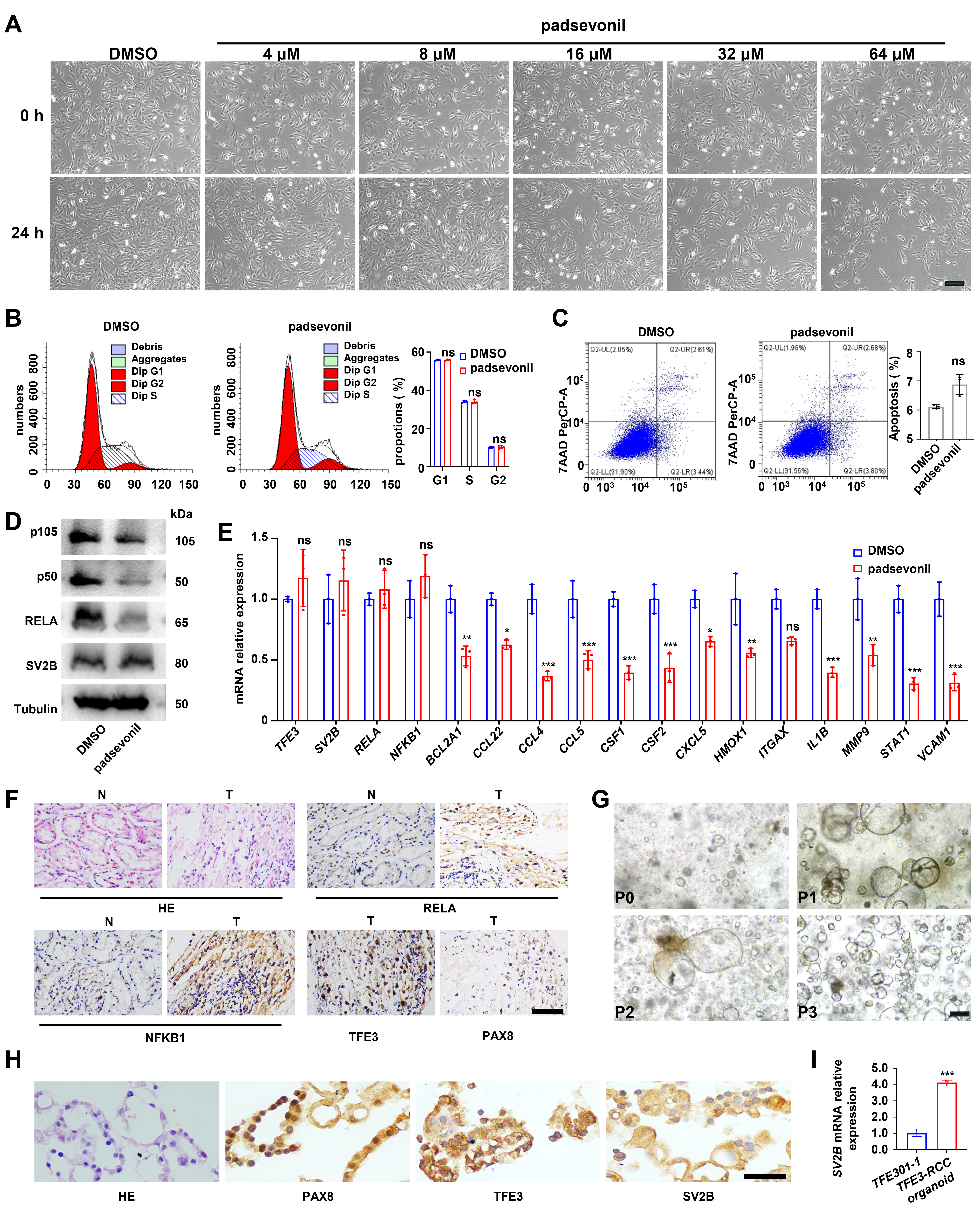


**Figure S10 Padsevonil can inhibit TFE3-RCC progression.**

**A**. Inhibition of cell proliferation became more pronounced with the increase in padsevonil concentration. **B-C**. Effect of 20 μM padsevonil on cell cycle (B) and apoptosis (C) in TFE301-1 cells. **D-E**. TFE301-1 cells treated with 20 μM padsevonil showed lower activation of NF-κB pathway compared to that treated with DMSO. **F-H**. A TFE3-RCC organoid was successfully established and passaged. **I.** *SV2B* expression in TFE301-1 cells and TFE3-RCC organoids. These experiments were replicated three times. Data are presented as the mean ± SD. Scale bars = 200 μm; ns, not significant; *, p < 0.05; **, p < 0.01; ***, p < 0.001.


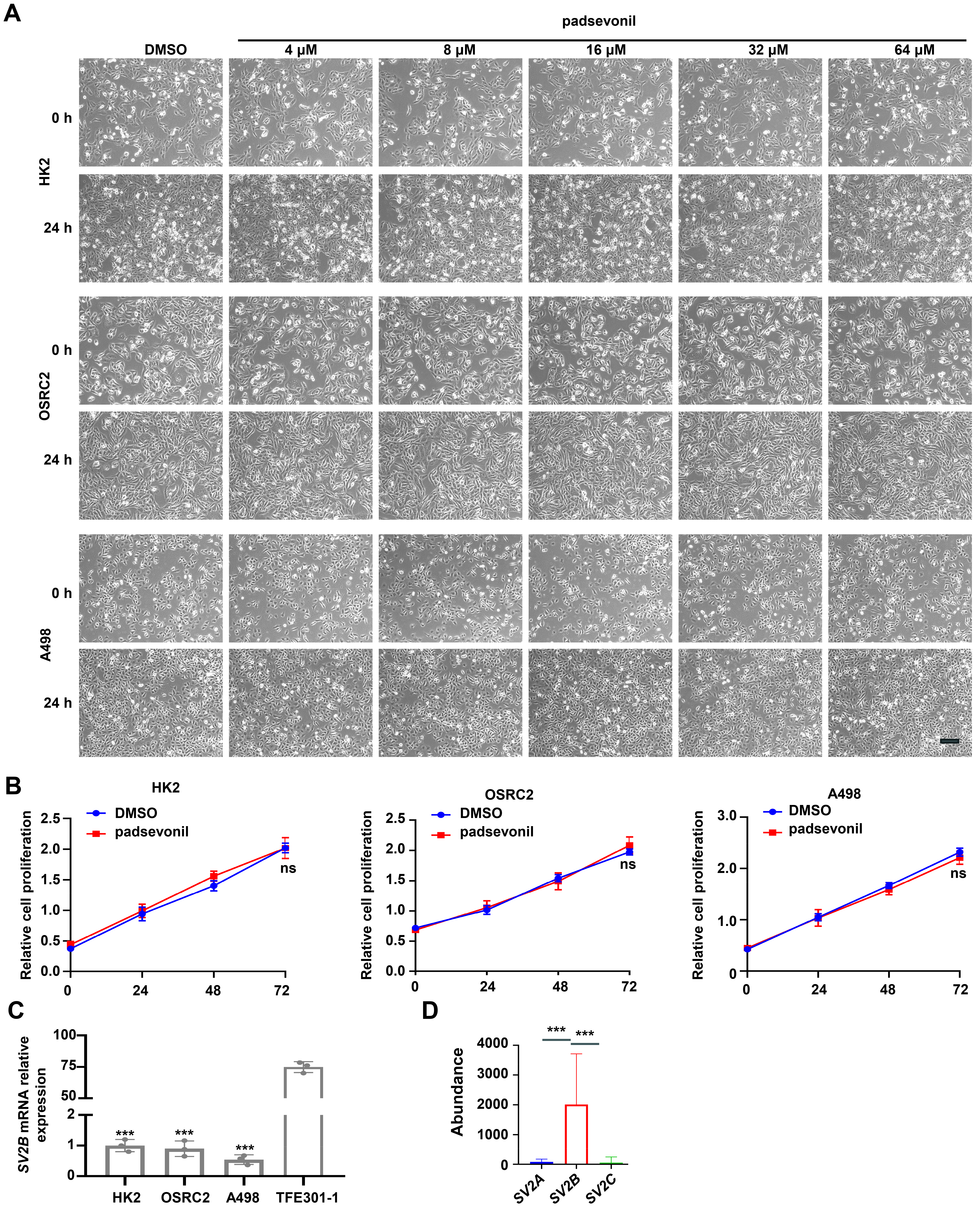


**Figure S11 No significant inhibitory effect of padsevonil on HK2, OSRC2, and A498 cells are observed.**

**A**. Padsevonil at low concentrations (4 μM) or high concentrations (64 μM) had no obvious effect on HK2, OSRC2, and A498 cell proliferation. **B**. 20 μM padsevonil had no obvious effect on HK2, OSRC2, and A498 cell proliferation. **C.** *SV2B* expression in HK2, OSRC2, A498 and TFE301-1 cells. **D.** *SV2A*, *SV2B* and *SV2C* expression in TFE3-RCC tissues using the GEO database**.** These experiments were replicated three times. Scale bars = 200 μm; ns, not significant.


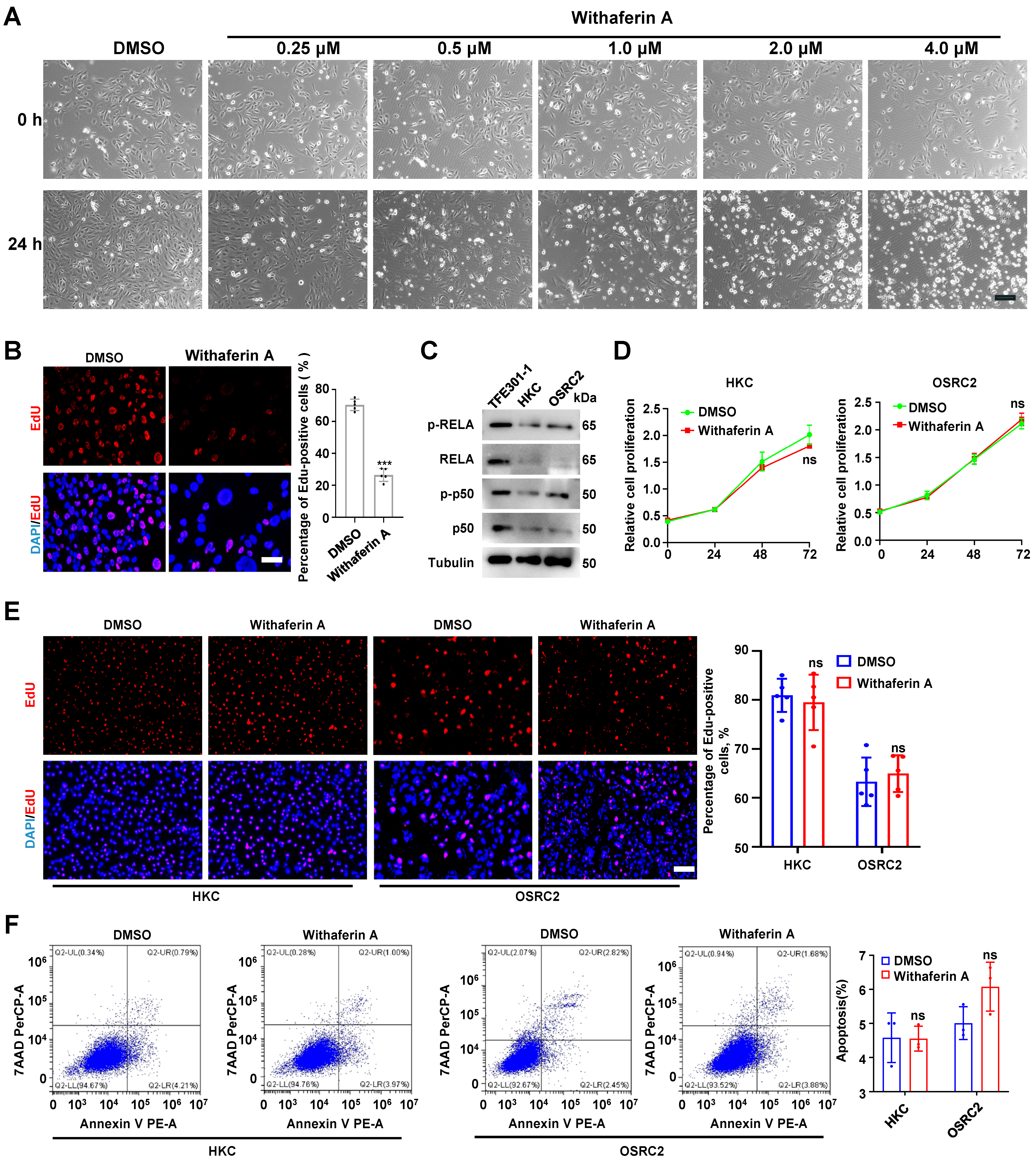


**Figure S12 Withaferin A inhibits TFE3-RCC progression.**

**A**. Inhibition of cell proliferation became more pronounced with the increase in withaferin A concentration. **B**. 0.5 μM Withaferin A inhibited the proliferation of TFE301-1 cells. Scale bars = 40 μm. **C**. RELA and NFKB1 expression in TFE301-1, HKC, and OSRC2 cells. **D-F**. 0.5 μM had no obvious effect on HKC and OSRC2 cell proliferation and apoptosis. These experiments were replicated three times. Data are presented as the mean ± SD. Scale bars = 80 μm. ns, not significant; *, p < 0.05; ***, p < 0.001.

**Supplemental Tables**

**Table S1** Top thirty TFE3-RCC-upregulated genes ranked by adjusted p value from differential expression analysis of TFE3-RCC vs. ARTs.

| ID | logFC | AveExpr | p.Value | adj.p.Val |
| --- | --- | --- | --- | --- |
| BHLHE41 | 4.247841308 | 9.900313293 | 6.08456E-31 | 2.09023E-26 |
| GPR143 | 5.378394521 | 9.104227616 | 2.00798E-25 | 3.44901E-21 |
| PKD1L2 | 6.299741419 | 10.53850824 | 5.92132E-23 | 6.78051E-19 |
| **NMRK2** | 7.58449982 | 9.700544115 | 5.64821E-21 | 4.85082E-17 |
| LDLRAD3 | 2.883485474 | 8.518798986 | 1.05246E-20 | 7.23101E-17 |
| IRX6 | 4.814388321 | 7.991321013 | 3.47848E-20 | 1.9916E-16 |
| HABP2 | 5.637268529 | 10.36186272 | 1.29953E-19 | 6.37752E-16 |
| **SV2B** | 5.635521035 | 9.240997576 | 3.02075E-19 | 1.29715E-15 |
| TRIM63 | 6.834363396 | 8.813028054 | 2.80283E-18 | 8.0238E-15 |
| PIM1 | 2.188723635 | 9.834928677 | 3.89962E-18 | 1.03049E-14 |
| CHST11 | 2.869977589 | 8.879028132 | 5.16214E-18 | 1.26668E-14 |
| SCG5 | 4.677878506 | 4.826535911 | 6.40692E-18 | 1.46731E-14 |
| RAP2B | 1.686549772 | 9.391165365 | 9.4816E-18 | 2.03576E-14 |
| SPSB1 | 2.495807635 | 8.867879907 | 1.01255E-17 | 2.04613E-14 |
| LILRB4 | 2.856781518 | 6.229674139 | 1.22904E-17 | 2.34563E-14 |
| PVT1 | 2.957404738 | 7.039605125 | 1.48369E-17 | 2.68259E-14 |
| TNFRSF10B | 1.251149263 | 9.843319606 | 1.93953E-17 | 3.33143E-14 |
| AEN | 1.591581258 | 7.777582487 | 3.40401E-17 | 5.56847E-14 |
| GPNMB | 4.759251428 | 12.52158223 | 4.88363E-17 | 7.29424E-14 |
| SLC15A4 | 1.846768987 | 8.969013637 | 9.74679E-17 | 1.33933E-13 |
| MMP14 | 2.074264452 | 11.0722358 | 1.10132E-16 | 1.40124E-13 |
| APOC1 | 4.897442668 | 6.995334382 | 4.77006E-16 | 5.46219E-13 |
| C2 | 3.615459656 | 7.93521592 | 5.10245E-16 | 5.63256E-13 |
| SLC41A2 | 1.758940323 | 7.682248983 | 5.24676E-16 | 5.63256E-13 |
| MSC | 3.838995184 | 8.038934826 | 5.6744E-16 | 5.90705E-13 |
| GCNT3 | 3.644201058 | 7.513365182 | 7.78572E-16 | 7.86656E-13 |
| BIRC7 | 6.232878557 | 7.720033663 | 1.30144E-15 | 1.27738E-12 |
| CORO1C | 1.771798426 | 10.1286128 | 1.89863E-15 | 1.76281E-12 |
| SH3PXD2B | 3.348440492 | 10.32227753 | 2.93662E-15 | 2.5901E-12 |
| SIRPA | 1.678992772 | 10.55912766 | 2.94047E-15 | 2.5901E-12 |

**Table S2** Top thirty TFE3-RCC-upregulated genes ranked by adjusted p value from differential expression analysis of TFE3-RCC vs. KIRP.

| ID | logFC | AveExpr | *p*.Value | adj.*p*.Val |
| --- | --- | --- | --- | --- |
| ATP11AUN | 6.821517198 | -4.178540495 | 3.58974E-44 | 8.12788E-40 |
| **NMRK2** | 12.04491298 | -1.676324322 | 4.47099E-43 | 5.06161E-39 |
| RNA5SP40 | 4.109275086 | -5.771703407 | 4.11927E-39 | 3.10895E-35 |
| RDH8 | 5.70952678 | -5.483473073 | 8.28084E-39 | 4.68737E-35 |
| SCRT1 | 5.876934766 | -3.543929839 | 2.83101E-36 | 1.28199E-32 |
| EPHA5 | 7.010271455 | -5.067923394 | 5.36295E-35 | 2.0238E-31 |
| ANKRD30B | 5.816876025 | -5.000995772 | 1.11543E-34 | 3.60793E-31 |
| SNCB | 6.877982894 | -4.304902759 | 4.32828E-33 | 1.22501E-29 |
| TRPM7 | 2.168863226 | 5.480912802 | 1.70463E-32 | 4.28848E-29 |
| PLK5 | 6.223598065 | -4.081158711 | 3.26483E-29 | 7.39223E-26 |
| PDCD4 | 1.771279507 | 5.64077428 | 3.70734E-29 | 7.63105E-26 |
| SSTR2 | 4.157334176 | 0.501605995 | 3.88933E-28 | 7.33852E-25 |
| HHATL | 8.390957725 | -2.800570643 | 8.25403E-28 | 1.4376E-24 |
| RNU1-47P | 4.96106994 | -4.455102003 | 6.17977E-27 | 9.84477E-24 |
| PRKAG2 | 2.148843848 | 4.744802757 | 6.52202E-27 | 9.84477E-24 |
| DLL1 | 4.214975934 | 1.37106699 | 1.31561E-26 | 1.86175E-23 |
| CATSPERG | 3.880598061 | 1.835722044 | 4.13823E-26 | 5.51164E-23 |
| RHOXF1P1 | 5.958291711 | -4.848349417 | 4.69798E-26 | 5.90954E-23 |
| KLK15 | 5.831876113 | -5.209791598 | 3.52244E-25 | 3.98775E-22 |
| **SV2B** | 5.988796364 | 1.147067143 | 4.28168E-25 | 4.61647E-22 |
| CAVIN4 | 3.427141288 | -1.824957946 | 1.95836E-24 | 2.0155E-21 |
| LDLRAD3 | 2.270664928 | 3.868545529 | 4.89376E-24 | 4.81759E-21 |
| KCNA1 | 3.415236752 | -5.585557516 | 6.45911E-24 | 6.09363E-21 |
| TRIM67 | 5.130965291 | -2.040520392 | 9.18967E-24 | 8.3229E-21 |
| ASCL1 | 4.834917813 | -5.177467496 | 1.87528E-23 | 1.63308E-20 |
| C21orf91 | 2.157194708 | 3.248668935 | 4.20414E-23 | 3.52556E-20 |
| PART1 | 6.313396866 | -4.131580128 | 4.91424E-23 | 3.92084E-20 |
| ACOT12 | 5.837194502 | -4.477625955 | 5.02183E-23 | 3.92084E-20 |
| AGPAT3 | 1.58741976 | 7.153326905 | 2.248E-22 | 1.69664E-19 |
| SCN4B | 3.58408617 | 1.593943089 | 2.39629E-22 | 1.75022E-19 |

**Table S3** Top thirty TFE3-RCC-upregulated genes ranked by adjusted p value from differential expression analysis of TFE3-RCC vs. KIRC.

| ID | logFC | AveExpr | *p*.Value | adj.*p*.Val |
| --- | --- | --- | --- | --- |
| **NMRK2** | 14.10419443 | -3.947527048 | 1.6635E-77 | 1.91868E-73 |
| SCRT1 | 6.848538492 | -4.619784051 | 2.06821E-56 | 1.19274E-52 |
| TRIM67 | 6.381715917 | -3.371968957 | 1.43301E-53 | 5.50944E-50 |
| PLK5 | 7.093239743 | -5.06528426 | 1.61189E-51 | 4.6479E-48 |
| EPHA5 | 7.181660163 | -5.388989419 | 1.11674E-49 | 1.24048E-45 |
| TRIM63 | 8.691202532 | -2.210597339 | 2.35776E-48 | 5.43889E-45 |
| TSPAN10 | 5.030596403 | 0.671150908 | 5.73778E-47 | 1.10299E-43 |
| ATP11AUN | 6.7248359 | -4.23426166 | 3.54679E-47 | 1.96989E-43 |
| CYP17A1 | 7.498935066 | -0.090325086 | 7.75899E-45 | 2.87289E-41 |
| CATSPERG | 4.157163187 | 1.480960461 | 1.11373E-41 | 3.09283E-38 |
| SNCB | 7.080027886 | -4.652931569 | 3.1381E-41 | 5.17069E-38 |
| CFAP61 | 4.850825397 | -1.716458973 | 1.11038E-40 | 2.46682E-37 |
| SEPHS2 | 2.549459269 | 6.948728852 | 1.51333E-39 | 2.18185E-36 |
| CDH17 | 7.368299419 | -1.345620546 | 2.99889E-39 | 5.55195E-36 |
| **SV2B** | 7.025786543 | 0.005044558 | 3.7774E-38 | 4.84095E-35 |
| LGI3 | 6.723798532 | -3.312506158 | 3.29973E-37 | 5.23619E-34 |
| NAGLU | 2.134408513 | 5.61245349 | 5.65156E-37 | 6.51851E-34 |
| FAM124A | 3.774428618 | 2.410891042 | 5.29533E-37 | 7.35257E-34 |
| RHOBTB2 | 2.134622431 | 4.705020236 | 2.46343E-36 | 2.58302E-33 |
| ACOT12 | 5.857554977 | -4.625754355 | 1.20306E-35 | 1.48485E-32 |
| RDH8 | 5.531541345 | -5.435535472 | 4.20714E-35 | 4.04376E-32 |
| INSM1 | 4.674328406 | -5.455463746 | 6.672E-35 | 6.73751E-32 |
| NDRG4 | 3.922747144 | 1.865549102 | 1.49772E-34 | 1.32882E-31 |
| SLC51B | 4.495139919 | 0.882661144 | 1.82079E-33 | 1.50007E-30 |
| ATP4B | 5.126032916 | -4.613287309 | 4.94986E-33 | 4.58192E-30 |
| LINC00410 | 3.555752006 | -5.920406635 | 3.7461E-32 | 3.2009E-29 |
| ASTN1 | 7.010154231 | -2.723726549 | 4.50753E-32 | 3.5764E-29 |
| ABCB5 | 8.009501673 | -3.164220316 | 2.75231E-31 | 2.03818E-28 |
| KLK4 | 9.614565678 | -4.15237466 | 2.84381E-30 | 1.97431E-27 |
| GPAT4 | 1.645849808 | 6.238554539 | 4.61544E-30 | 3.01578E-27 |

**Table S4** Fusion types of 14 TFE3-RCC cases from TCGA.

| ID | *SV2B* relative expression | Fusion Types |
| --- | --- | --- |
| TCGA.2Z.A9JO.01A.11R.A42S.07 | 8.03833433750315 | U2AF2-TFE3 Fusion |
| TCGA.6D.AA2E.01A.11R.A37O.07 | 6.98263185660043 | MED15-TFE3 Fusion |
| TCGA.AK.3456.01A.02R.1325.07 | 8.15025664405823 | SFPQ-TFE3 Fusion |
| TCGA.B0.5705.01A.11R.1541.07 | 1.90361896798288 | SFPQ-TFE3 Fusion |
| TCGA.B8.5546.01A.01R.1541.07 | 8.64484543661736 | SFPQ-TFE3 Fusion |
| TCGA.BP.4756.01A.01R.1289.07 | 8.46835408711178 | SFPQ-TFE3 Fusion |
| TCGA.BP.4758.01A.01R.1289.07 | 1.89824599004905 | SFPQ-TFE3 Fusion |
| TCGA.BQ.5882.01A.11R.1592.07 | 7.80005231624485 | PRCC-TFE3 Fusion |
| TCGA.BQ.5887.01A.11R.1965.07 | 7.53895260720643 | PRCC-TFE3 Fusion |
| TCGA.BQ.7050.01A.11R.1965.07 | 7.93513055369321 | PRCC-TFE3 Fusion |
| TCGA.CJ.5681.01A.11R.1541.07 | 8.5215855971413 | KHSRP-TFE3 Fusion |
| TCGA.G7.7501.01A.11R.2204.07 | 7.7516362306898 | SFPQ-TFE3 Fusion |
| TCGA.J7.8537.01A.11R.2404.07 | 4.22445843456077 | DVL2-TFE3 Fusion |
| TCGA.SX.A7SO.01A.11R.A355.07 | 8.05145512435039 | SFPQ-TFE3 Fusion |

**Table S5** Primer sequences for ChIP-qPCR primers.

| Primers | Forward primers | Reverse primers |
| --- | --- | --- |
| Ctrl Primer | TACTAGCGGTTTTACGGGCG | TCGAACAGGAGGAGCAGAGAGCGA |
| Primers 1 | CGGAGGTTGAGGCTGTGAT | CGCAAGGACTTCAACTTCTCA |
| Primers 2 | AAAAGATGAGGGTGGAGGGG | GGAACTCCTGAGCTCAAGTG |
| Primers 3 | GAAGCCACACCCAAAAGAGT | TGTAACCACCACTCTGACCC |
| Primers 4 | CAGCCTGGCCAACATTTCAA | CCTCCACCTCCCAGATTCAA |
